# Antigen specificities and proviral integration sites differ in HIV-infected cells by timing of antiretroviral treatment initiation

**DOI:** 10.1172/JCI159569

**Published:** 2024-06-04

**Authors:** Jaimy Joy, Ana Gervassi, Lennie Chen, Brent Kirshenbaum, Sheila Styrchak, Daisy Ko, Sherry McLaughlin, Danica Shao, Ewelina Kosmider, Paul T. Edlefsen, Janine Maenza, Ann C. Collier, James I. Mullins, Helen Horton, Lisa M. Frenkel

**Affiliations:** 1 Center for Global Infectious Disease Research, Seattle Children’s Research Institute, Seattle, Washington, USA.; 2 Department of Microbiology, University of Washington, Seattle, Washington, USA.; 3 Center for Infectious Disease Research, Seattle, Washington, USA.; 4 Vaccine and Infectious Disease Division, Fred Hutchinson Cancer Research Center, Seattle, Washington, USA.; 5 Department of Medicine,; 6 Department of Global Health,; 7 Department of Pediatrics, and; 8 Department of Laboratory Medicine and Pathology, University of Washington, Seattle, Washington, USA.

**Keywords:** AIDS/HIV, Virology, Antigen, Cytokines, T cells

## Abstract

Despite effective antiretroviral therapy (ART), persons living with HIV harbor reservoirs of persistently infected CD4^+^ cells, which constitute a barrier to cure. Initiation of ART during acute infection reduces the size of the HIV reservoir, and we hypothesized that in addition, it would favor integration of proviruses in HIV-specific CD4^+^ T cells, while initiation of ART during chronic HIV infection would favor relatively more proviruses in herpesvirus-specific cells. We further hypothesized that proviruses in acute ART initiators would be integrated into antiviral genes, whereas integration sites (ISs) in chronic ART initiators would favor genes associated with cell proliferation and exhaustion. We found that the HIV DNA distribution across HIV-specific versus herpesvirus-specific CD4^+^ T cells was as hypothesized. HIV ISs in acute ART initiators were significantly enriched in gene sets controlling lipid metabolism and HIF-1α–mediated hypoxia, both metabolic pathways active in early HIV infection. Persistence of these infected cells during prolonged ART suggests a survival advantage. ISs in chronic ART initiators were enriched in a gene set controlling EZH2 histone methylation, and methylation has been associated with diminished long terminal repeat transcription. These differences that we found in antigen specificities and IS distributions within HIV-infected cells might be leveraged in designing cure strategies tailored to the timing of ART initiation.

## Abstract 2

The timing of antiretroviral treatment initiation affects antigen specificities and integration sites of HIV-infected cells.

## Introduction

Antiretroviral therapy (ART) can effectively suppress HIV replication, but maintenance of virologic suppression requires that persons living with HIV adhere to the prescribed medicines over their lifetime, as current ART does not cure the infection. Initiation of ART during acute HIV infection minimizes the size of an individual’s HIV reservoir (1–5), defined variably as replication-competent (transcriptionally active or latent) proviruses or by HIV DNA that persists during ART. The HIV reservoir slowly decreases in size with time, but it persists for many years and nearly always leads to viral rebound following ART cessation (6–8). Cells that contain the HIV reservoir often undergo clonal proliferation, purportedly from homeostatic proliferation, antigen stimulation, and/or from HIV integration site–promoted (IS-promoted) proliferation/survival (reviewed in refs. 9–11).

HIV-specific CD4^+^ T cells, upon encountering cognate antigens during acute HIV infection, are activated, preferentially infected, and undergo several rounds of proliferation (12). During untreated chronic HIV infection, CD4^+^ T cells specific for human herpesviruses, including Epstein-Barr virus (EBV), cytomegalovirus (CMV), herpes simplex viruses types 1 and 2 (HSV1 and -2), as well as for HIV and other infections, become activated and serve as targets for HIV infection (13). Because antigen-driven clonal proliferation appears to be a major contributor to HIV persistence (14), targeting the antigen specificities of HIV-infected CD4^+^ T cells has been proposed to reverse proviral latency as part of a “shock-and-kill” strategy (15).

Evidence suggests that HIV integration into certain cancer-related genes, such as *STAT5B* and *BACH2*, promotes clonal expansion of infected cells (11, 16, 17). IS-mediated clonal expansion can be driven by the HIV 5′ long terminal repeat (LTR) promoting expression of the gene in which the provirus has integrated (18, 19). Furthermore, transcriptome analysis of HIV-infected single cells revealed that HIV can drive high aberrant host gene transcription downstream of the IS, which can in turn induce aberrant splicing, intron retention, and cryptic exon activation at the IS (20). Thus, beyond insertional activation, the IS can drive aberrant host gene expression, which may promote IS-mediated clonal expansion.

We hypothesized that individuals who initiate ART in acute infection have ISs concentrated in genes involved in antiviral pathways and individuals who initiate ART in chronic infection have ISs distributed across many different genes, especially those involved in proliferation and T cell exhaustion. Our hypotheses were based on the reasoning that the immediate immune response to HIV infection will activate HIV-specific CD4^+^ T cells to initiate an antiviral response and HIV will infect and integrate into these genes, and during untreated chronic HIV infection, herpesviruses will recur and HIV-specific CD4^+^ T cells will express high levels of exhaustion markers, such as Tim3 and PD-1, compared with healthy controls (21, 22), and HIV will infect and integrate into expressed genes.

We reasoned that both antigen specificity and functional properties of genes harboring an IS could be exploited in HIV cure strategies. To provide insights into this possibility, we compared HIV DNA load in HIV-specific and herpesvirus-specific CD4^+^ T cells and gene sets with ISs between ART-suppressed individuals who initiated ART during acute or chronic HIV infection.

## Results

**Figure 1:**
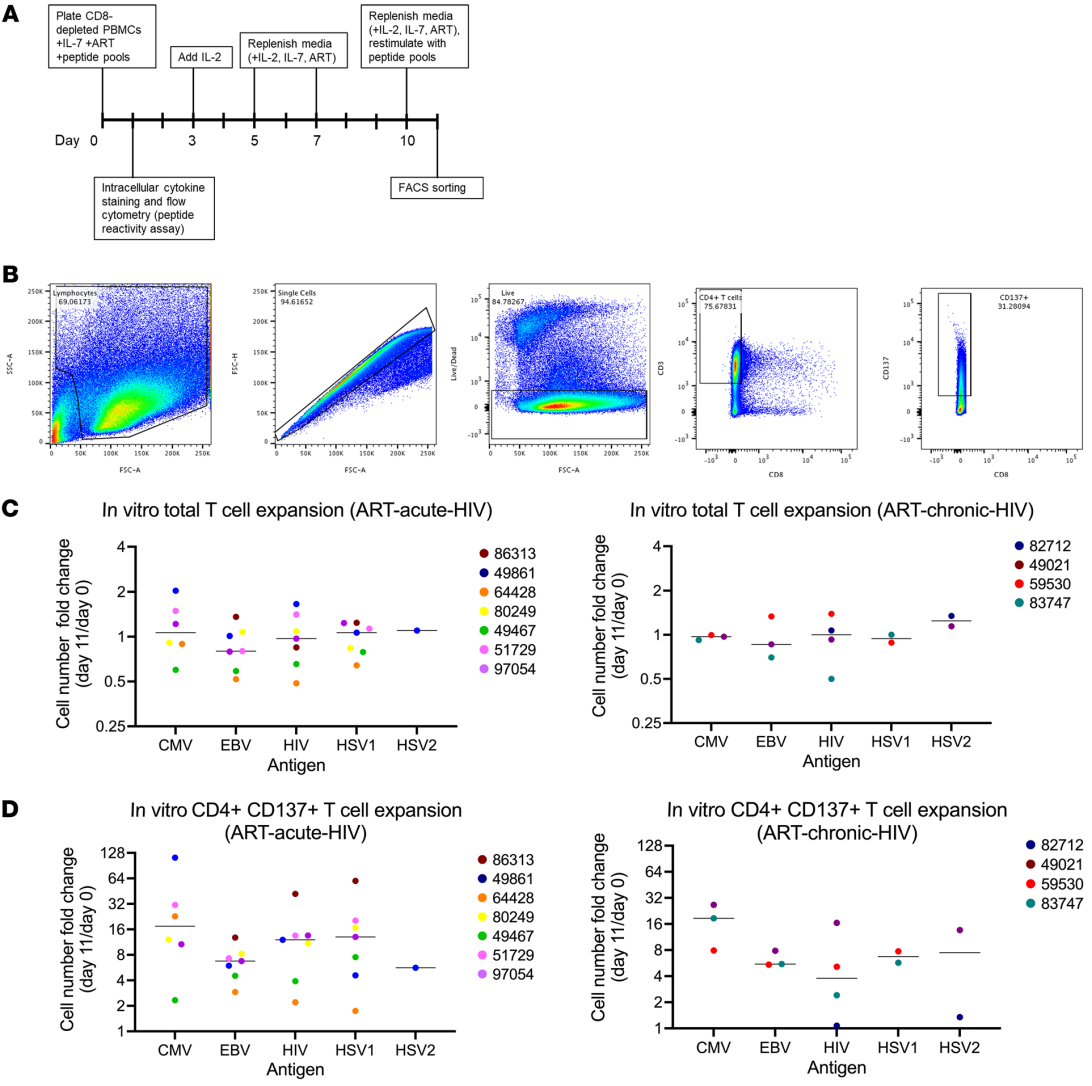
Peptide antigen stimulation and CD3^+^CD8^–^CD137^+^ cell expansion in vitro. (**A**) Experimental schema. CD8^+^ T cell–depleted PBMCs were incubated with anti-CD3/anti-CD28 Dynabeads (positive control), media alone (negative control), or peptide antigens derived from HIV, CMV, EBV, HSV1, or HSV2 (see Table 2) for 10 days, all with efavirenz, raltegravir, and IL-7 (ART). On day 3, IL-2 was added to the cultures. On days 5, 7, and 10, the media, ART, and growth factors were replenished. On day 10, cells were restimulated with peptide antigens to upregulate activation markers for cell sorting. Following 24 hours of restimulation, cells were harvested for intracellular cytokine staining and flow cytometry to assess reactivity to each antigen, i.e., “antigen discovery.” Cells were stained for CD3, CD8, and CD137 for cell sorting. CD8^+^ T cell–depleted PBMCs were plated on day 0, cultured as shown in **A**, and enumerated on day 11. (**B**) Representative flow cytometry plots for cell sorting based on CD3^+^CD8^–^CD137^+^ cell surface expression. (**C**) Fold change of total cells on day 11 versus day 0 and (**D**) fold change of CD3^+^CD8^–^CD137^+^ cells on day 11 versus day 0 are shown for individuals who initiated ART-chronic-HIV in left panels and for individuals who initiated ART-chronic-HIV in right panels. Horizontal lines indicate median of data points.

Male participants in the Seattle Primary Infection Cohort (23–25) who initiated ART within 6 weeks (*n* = 7) (ART-acute-HIV) or after more than 6 months from the estimated date of HIV infection (*n* = 4) (ART-chronic-HIV) were studied (Table 1). The HIV DNA concentrations and ISs were compared by antigen specificities of participants’ infected CD4^+^ T cells from blood specimens collected after a median 10.1 years (range 2.4–17.1) of ART suppression. Initially, we measured HIV DNA in antigen-specific cells after 24 hours of peptide antigen stimulation. However, after sorting, the number of CD137^+^ cells was limited, and the HIV DNA measurements were near or below the limit of detection. Therefore, virus-specific CD4^+^ cells were expanded by stimulation of CD8^+^ T cell–depleted PBMCs with peptide antigen pools in media supplemented with IL-7, raltegravir, and efavirenz (day 0), IL-2 (day 3), and restimulated on day 10. Twenty-four hours after restimulation, cells were sorted based on CD137 surface expression (Figure 1, A and B, and Supplemental Tables 1 and 2; supplemental material available online with this article; https://doi.org/10.1172/JCI159569DS1). Only one activation marker (CD137) was utilized, as the measurements were performed prior to the reporting of the activation-induced marker (AIM) assay using 2 markers (26). Serologic and cellular responses to HIV and herpesviruses were assessed for each participant (Table 2), with peptide antigen pools screened for IFN-γ, TNF-α, and IL-2 reactivity (see Methods and Supplemental Figure 1). The total number of live cells after restimulation was largely unchanged from the starting input (Figure 1C), likely due to the small number of cells activated to proliferate by each antigen. The expansion of CD8^–^, antigen-specific CD3^+^CD137^+^ cells varied somewhat by participant and viral antigens tested (Figure 1D and Supplemental Table 3; statistical analyses were not performed due to insufficient power from sparse data, as some participants were not uniformly reactive to herpesviruses).

**Figure 2:**
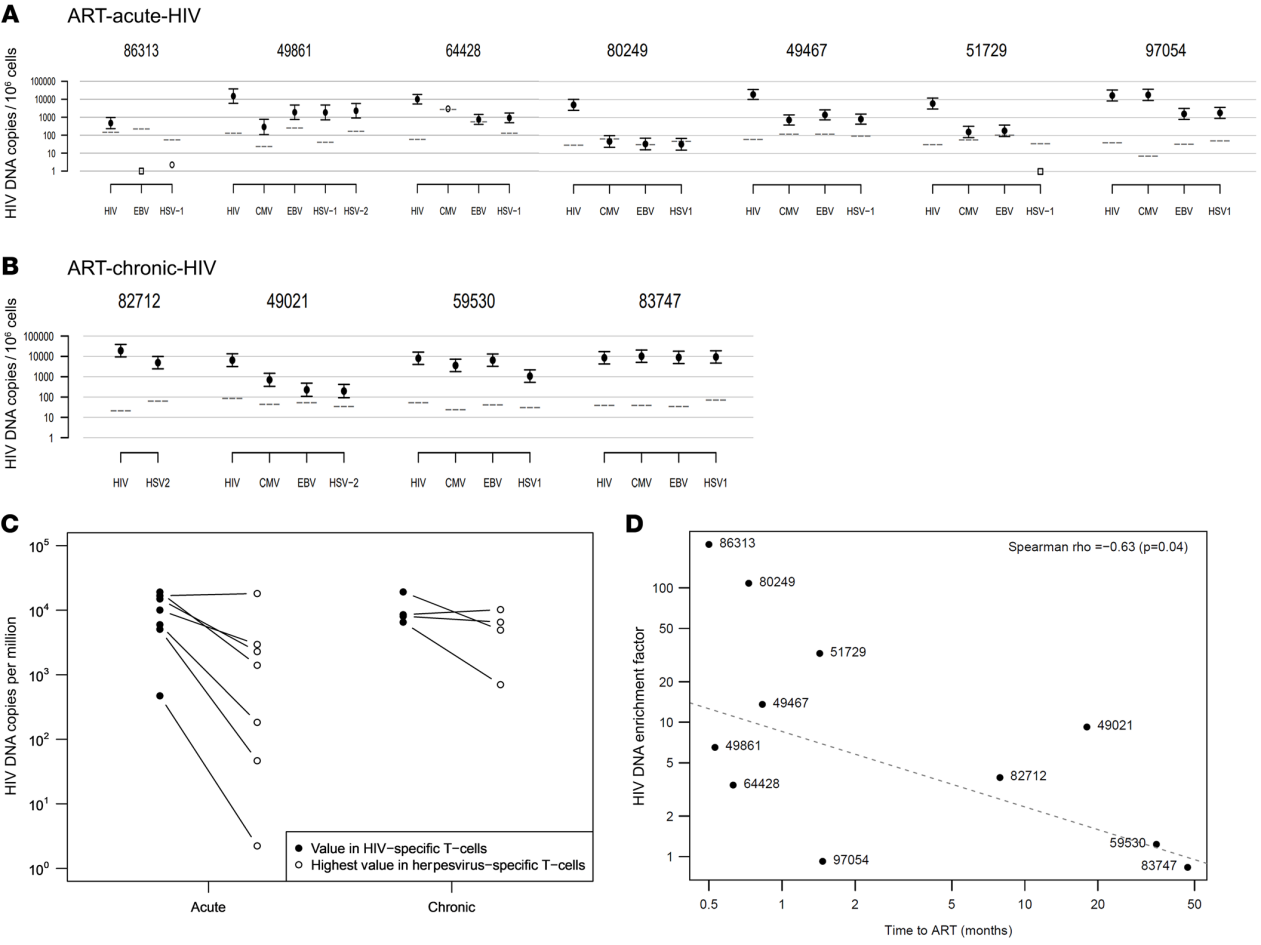
HIV DNA levels in antigen-specific CD4^+^ T cells of participants initiating ART during acute or chronic HIV infection. (**A** and **B**) HIV DNA levels (*y* axis) in CD4^+^ T cells reactive (CD3^+^CD8^–^CD137^+^) to HIV or various herpesviruses (*x* axis) 11 days after peptide antigen stimulation are shown for participants initiating ART during acute (**A**) or chronic (**B**) HIV infection. The ART-acute-HIV cohort (*n* = 7) was defined as 1.5 months or less between estimated time of infection and ART initiation and the ART-chronic-HIV cohort (*n* = 4) was defined as more than 6 months between estimated time of infection and ART initiation. HIV DNA loads were measured by amplification of HIV genomic regions (*env*, *gag*, *LTR*) multiplexed with the human gene transferrin gene (hTFR) by qPCR. HIV DNA is represented as LTR copies per 10^6^ cells. Each solid circular symbol is the mean log(LTR/hTFR) of 2 replicate measures, with bars indicating 2 standard errors. When only one replicate was above the limit of detection, an open circular symbol is shown, and when neither replicate was above the limit of detection, an open square at 1 is shown. Dotted lines represent equivalent of 1 copy of HIV per million cells (see Methods). (**C**) Comparison of within-participant differences of HIV DNA in HIV-specific CD4^+^ T cells versus highest HIV DNA among herpesviruses-specific CD4^+^ T cells across ART-acute-HIV and ART-chronic-HIV groups. Wilcoxon’s *P* = 0.79. (**D**) The HIV DNA enrichment factor (*y* axis, defined as the HIV DNA load in HIV-specific cells divided by the herpesviruses-specific cells with the greatest HIV DNA load) versus time from acute HIV infection to ART initiation (*x* axis); Spearman’s δ (*P* = 0.04) shows a negative correlation.

HIV DNA in antigen-specific CD4^+^ T cells following the 11-day antigen stimulation was measured using the viral open reading frame detection assay (VODA) (Figure 2A). HIV DNA appeared disproportionately higher in HIV-specific compared with herpesvirus-specific CD4^+^ T cells for participants in the ART-acute-HIV group, except in participant 97054, who had equal levels of HIV DNA in HIV-specific and CMV-specific T cells. Of note, this was the only participant in the ART-acute-HIV group who had plasma HIV RNA detected during ART (50–120 copies/mL were detected on 4 of 5 determinations in 2012–2014) (Supplemental Figure 2). In the ART-chronic-HIV group, HIV DNA appeared to be relatively evenly distributed across HIV-specific and herpesvirus-specific cells in 3 of 4 participants (Figure 2B), with participant 49021 having more HIV DNA in HIV-specific compared with his herpesvirus-specific cells (Figure 2B). This participant’s CD4^+^ cell count remained in the normal range during the 18 months he was untreated prior to ART initiation (Supplemental Figure 2), which may have limited recurrences of herpesviruses compared with others in the ART-chronic-HIV group, whose CD4^+^ counts decreased to between 200 and 400 cells/μL prior to ART initiation. To further assess the relationship between the time interval spanning from HIV acquisition to ART initiation and the proportion of proviruses in HIV-specific cells, we compared the quantity of HIV DNA in HIV-specific T cells versus herpesvirus-specific T cells and found that the shorter this interval of time, the greater the enrichment of HIV DNA in HIV-specific cells (Figure 2, C and D) (Spearman’s δ = –0.63, *P* = 0.04).

We considered that higher levels of HIV DNA detected in HIV-specific cells after antigen stimulation may reflect relatively greater levels of proliferation by the HIV-specific cells during the 11-day in vitro antigen stimulation instead of differences between the timing of ART initiation. To evaluate this possibility, we compared CD4^+^ T cell proliferation in response to peptide antigens using a carboxyfluorescein succinimidyl ester (CFSE) dilution assay (Supplemental Figure 3). Across the ART-acute-HIV and ART-chronic-HIV groups, proliferation of HIV-specific cells exceeded that of herpesvirus-specific cells in only one (participant 97054) who had increased proliferation in response to HIV accessory protein viral protein R (Vpr). In all other individuals, proliferation of HIV-specific cells appeared similar or less than herpesviruses-specific cells, with apparent increased proliferation in response to EBV nuclear antigen 3C (EBNA3C) in participant 64428 and higher proliferation in response to pp65 in participants 86313 and 59530. Overall similar levels of proliferation between cells stimulated with HIV or herpesvirus antigens suggests that the relatively higher levels of HIV DNA detected in HIV-specific cells reflect the in vivo levels of HIV DNA in the participants and is not attributable to unequal cell expansion in culture.

**Figure 3:**
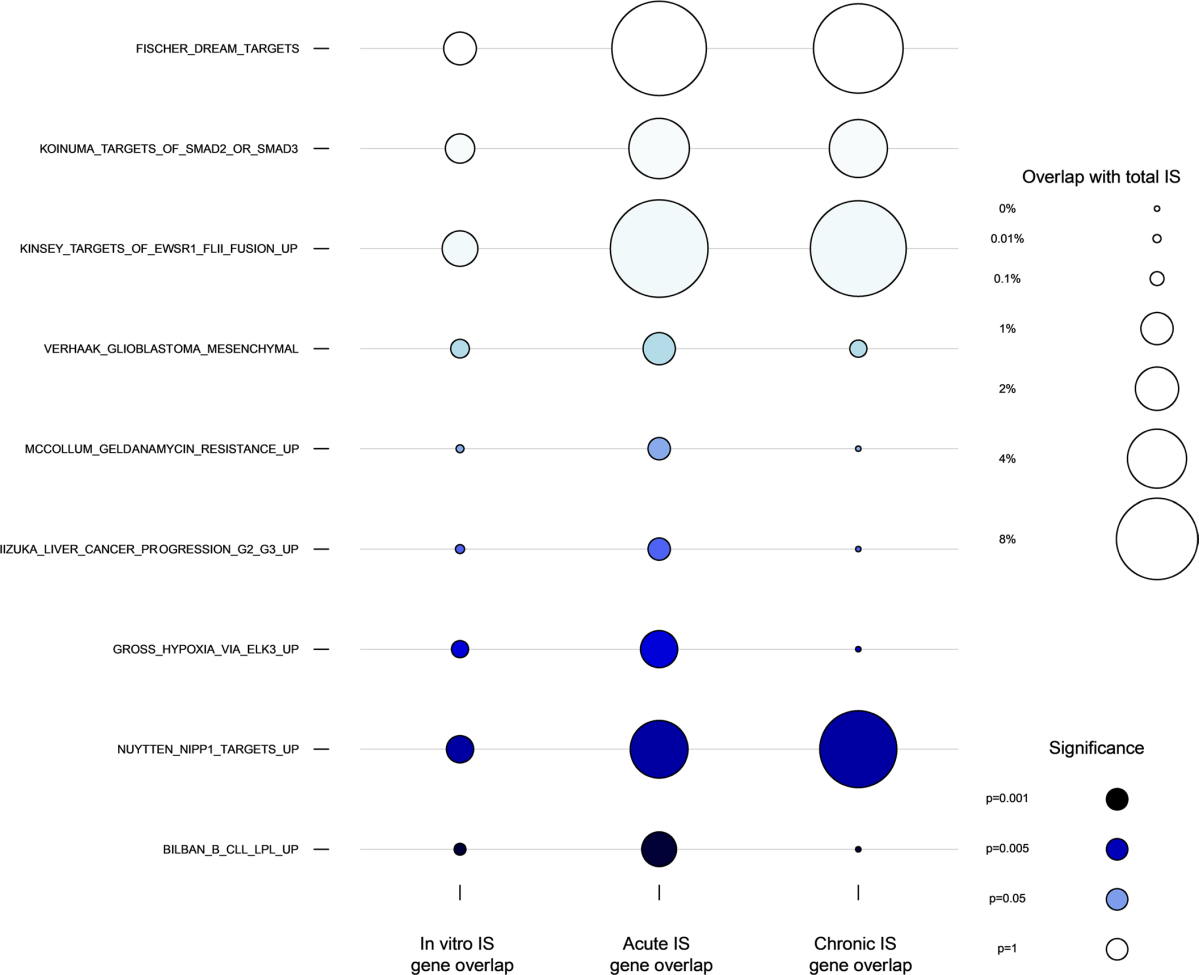
HIV IS gene set enrichment analysis comparing ISs in gene pathways (GSEA) from in vitro acutely HIV-infected primary CD4^+^ T cells, ART-acute-HIV, and ART-chronic-HIV groups. Three columns of bubbles represent the proportion of HIV ISs in the 9 gene sets (see Supplemental Table 4) from acute in vitro HIV infections of cells (leftmost column) (11, 27) compared to those we detected in the ART-acute-HIV and ART-chronic-HIV IS (center and rightmost columns). The size of the bubble indicates the magnitude of overlap between the gene set and unique ISs in each group and the color of the bubble represents the statistical significance of the acute versus chronic comparison. In vitro ISs are included in this plot to highlight the statistically significant difference in representation of ISs in the gene set in either ART-acute-HIV or ART-chronic-HIV versus in vitro (see Supplemental Table 4), which implies selection for HIV ISs in these gene sets over time on ART.

To evaluate our hypothesis that the gene pathways harboring integrated HIV differ between the ART-acute-HIV versus ART-chronic-HIV groups, ISs were derived (16) from negatively selected CD4^+^ T cells and compared to a set of approximately 66,000 ISs derived from in vitro infection of unstimulated primary CD4^+^ T cells infected with HIV-1 strain BaL (HIV-1_BaL_) and cultured for 48 hours (11), and primary resting CD4^+^ T cells infected with HIV NL4-3 for 96 hours (27) (referred to as “in vitro IS”) (see Methods). To identify gene sets associated with persistence of the reservoir during ART, we separately compared 1,083 unique ISs from the ART-acute-HIV and 632 unique ISs from ART-chronic-HIV groups to unique ISs derived from the aforementioned in vitro acutely infected cells across the 1,257 gene sets curated from MSigDB (28). Significant enrichment or depletion of HIV ISs was observed for 9 gene sets by Fisher’s exact test (11, 27–29) in one or both comparisons of the ART-acute-HIV group versus in vitro HIV–infected cells and/or the ART-chronic-HIV group versus in vitro HIV–infected cells (Supplemental Table 4). Among these 9 gene sets, HIV ISs in “BILBAN_B_CLL_LPL_UP,” “GROSS_HYPOXIA_VIA_ELK3_UP,” and “IIZUKA_LIVER_CANCER_PROGRESSION_G2_G3_UP” were significantly enriched in the ART-acute-HIV compared with the ART-chronic-HIV group and “NUYTTEN_NIPP1_TARGETS_UP” was significantly enriched in the ART-chronic-HIV compared with the ART-acute-HIV group (Supplemental Table 4). The proportion of ISs in nongenic or genic regions was not associated with the time interval between HIV infection and ART initiation in our cohort (Supplemental Figure 4). Finally, a comparison of the frequency of HIV ISs in the 9 gene sets by time to ART initiation to ISs in cells acutely infected in vitro confirmed significant selection for these gene sets during ART following ART initiation during acute infection (Figure 3).

## Discussion

This study of 11 men demonstrates that the interval between acquisition of HIV infection and ART initiation is roughly proportional to the size of the HIV reservoir despite years of ART suppression. Our findings include that the interval to ART initiation also appears to affect the CD4^+^ T cell antigen specificity and the gene pathways with HIV-infected cells that persist during ART. When ART was initiated during acute infection, HIV DNA appeared concentrated in HIV-specific CD4^+^ T cells, and when ART was initiated during chronic HIV infection, HIV DNA appeared more evenly distributed across HIV-specific and herpesviruses-specific CD4^+^ T cells. HIV ISs were significantly enriched in gene sets involved in the regulation of lipid metabolism and HIF-1α–mediated hypoxia in the ART-acute-HIV group, while ISs were enriched in enhancer of zeste homolog 2 (EZH2) histone methylation in the ART-chronic-HIV group. To our knowledge, this is the first study to compare antigen specificities and enrichment of HIV ISs across gene sets as a function of time between HIV infection and ART initiation. Our findings demonstrate that the reservoir appears relatively restricted to HIV-specific CD4^+^ T cells and enriched in genes of certain biological pathways when ART is initiated early in infection, suggesting that manipulation of these metabolic pathways may serve as avenues for therapeutic intervention in individuals who initiate ART during acute infection.

We believe this study is unique, as it compares reservoirs of ART-suppressed individuals who initiated ART during acute HIV infection, defined as less than 1.5 months from the estimated date of infection (25), or chronic infection, defined as more than 6 months. Previous studies that investigated antigen specificity of HIV-infected cells either evaluated only persons who initiated ART during chronic infection (14), or did not directly compare between those who initiated ART during acute or chronic infection (10, 12). The negative correlation that we observed between the duration of untreated HIV infection and enrichment of viral DNA in HIV-specific CD4^+^ T cells, taken together with others’ findings (10, 12, 30), support the hypothesis that acute HIV infection leads to infection of HIV-specific CD4^+^ T cells and active viral replication for 6 or more months leads to HIV infection of both HIV-specific CD4^+^ T cells and CD4^+^ T cells with other antigen specificities. Our finding of relatively high HIV DNA levels in HIV-specific CD4^+^ T cells of the ART-acute-HIV group despite years of suppressive ART demonstrates persistence of these infected cells or their clonal descendants. However, the distribution of HIV DNA across CD4^+^ T cells targeting HIV and multiple herpesviruses in the ART-chronic-HIV group align with previous reports of HIV-1 proviruses persisting in pp65- and gag-specific CD4^+^ T cells (10, 12), including clones of replication-competent proviruses in CMV-specific CD4^+^ T cells (14, 31).

Because our initial attempts to measure HIV DNA in antigen-specific cells after 24 hours of culture was unreliable, we expanded antigen-specific CD4^+^ T cells using peptide stimulation in culture over 10 days. Our CSFE dilution experiments suggest that HIV-specific and herpesviruses-specific T cells have few differences in in vitro proliferation in response to peptide antigens, and that the greater levels of HIV DNA were not due to preferential proliferation of HIV-specific CD4^+^ T cells. The higher proliferation in response to HIV Vpr, EBNA3C, and CMV pp65 detected on occasion could be due to central memory T cells, which are known to have greater proliferative capacities compared with effector memory T cells (32).

The gene pathways with HIV ISs that persist during ART appear to vary based on timing of ART initiation. ISs in the ART-acute-HIV group were enriched in genes associated with lipid metabolism and HIF-1α–mediated hypoxia, which are known to strongly influence functions of T lymphocytes (33, 34) and promote glycolytic metabolism in CD4^+^ T cells (35). CD4^+^ T cell activation increases both glycolysis and fatty acid metabolism to meet the energy needed for cell growth and effector functions and has been observed in acute HIV infection, with inhibition of glycolysis and fatty acid oxidation in vitro reducing HIV infection of cells (36). HIF-1α was recently shown to be hypermethylated in long-term nonprogressors and hypomethylated in individuals with detectable viral loads despite ART adherence (37). These results suggest that HIF-1α contributes to the ART suppression and possibly the persistence of infected cells. We speculate that proviruses integrated into genes controlling lipid metabolism and hypoxia-inducible factors could provide CD4^+^ T cells a survival advantage during years of ART suppression, perhaps through metabolic reprogramming toward a Treg phenotype (38). Gene editing studies are warranted to corroborate these findings and to assess the effects of proviral insertion on the expression of these genes.

ISs in the ART-chronic-HIV group were enriched in “NUYTTEN_NIPP1_TARGETS_UP,” which is associated with histone methylation by EZH2. EZH2 is an H3K27 methyltransferase that can lead to silencing of proviruses (39). The enrichment of this gene set in the HIV-chronic-ART group suggests that proviruses inserted in genes involved in epigenetic modifications mediated by EZH2 and other methyltransferases or deacetylases that maintain latency could lead to their increased persistence among individuals in the ART-chronic-HIV group.

Over long-term ART suppression, there is selection against transcriptionally active proviruses (40) and detection of intact proviruses in heterochromatic regions (41). Similarly, ISs in elite controllers are disproportionately increased in centromeric satellite DNA and other infrequently transcribed regions of the genome (42). While most ISs identified in our study are likely defective, defective proviruses can produce viral proteins and potentially elicit immune activation (43).

Limitations of this study include that only cisgender males were studied. It is uncertain whether antigen specificities of HIV-infected cells in females will demonstrate the same patterns, although in both males and females the amount of time on HIV treatment is associated with a smaller replication-competent HIV reservoir (44). Another limitation is that relatively few individuals were studied, especially in the HIV-chronic-ART group. The small number of individuals restricted the statistical power and limited the ability to compare expansion of antigen-specific T cells across antigens after peptide antigen stimulation, HIV DNA across antigens, and IS-enriched gene sets to the HIV-acute-ART group. Additionally, use of only CD137 to identify and sort virus-specific cells following peptide antigen stimulation may have reduced specificity of antigen-specific cells in our analyses, as others work shows that combinations of 2 activation markers (CD69 and CD40L [CD154], or OX40 and CD25, or OX40 and PD-L1 or 4-1BB), as outlined in the AIM assay (26), adds specificity to the detection of virus-specific cells. Another experimental limitation is that peptide stimulation may reactivate latent intact proviruses in vitro, which could (45, 46), but is not always, sufficient to induce cell death (47, 48). This study did not assess genomic or epigenetic features of integrated proviruses, including whether proviruses were intact or defective. Given that the former includes replication-competent viruses crucial for sustaining infection, and the observation that CMV-specific CD4^+^ T cell clones harboring replication-competent proviruses can be selected over time (14), such data may provide insights relevant to targeting proviruses for elimination.

Multiple studies have demonstrated the benefits of early ART initiation (reviewed in ref. 49), including smaller HIV DNA reservoirs (50), more rapid HIV DNA decay when ART is initiated earlier (51, 52, 53), and reduced loss of mucosal Th17 cells (54). Our results add to these reports and demonstrate that in addition to the total size of the HIV reservoir, early ART initiation shapes the antigen specificity of HIV-infected cells and biological pathways that harbor proviruses. Novel strategies are needed to cure HIV infection. Our findings suggest that cure regimens consider targeting antigen-specific cells and isolated biologic pathways.

## Methods

### Sex as a biological variable.

Sex as a biological variable was not investigated in our study since only one enrollee in our parent study of Primary HIV Infection in Seattle self-identified as a woman. This participant did not donate a leukapheresis specimen, which was required for this project.

### Study population.

This study included 11 participants, 7 of whom initiated ART during acute infection (ART-acute-HIV), defined as 1.5 months or less between estimated time of infection and ART initiation, and 4 of whom initiated ART during chronic infection (ART-chronic-HIV), defined as more than 6 months between estimated time of infection and ART initiation. All participants were males aged 39–61 (median age 53) from the Primary Infection Clinic cohort based in Seattle, which enrolled very few women (23–25). Participants were selected based on the following criteria: (a) ART-acute-HIV individuals initiated ART within 6 weeks and ART-chronic-HIV individuals initiated ART more than 6 months from estimated time of infection, (b) HIV replication was ART-suppressed for more than 2 years and plasma viral RNA levels that were undetectable (<50 copies/mL) or with rare viremias with HIV RNA up to 200 copies/mL, and (c) all had banked PBMC aliquots from leukapheresis (note: no participants identifying as women donated leukapheresis specimens). History of viral loads, CD4^+^ and CD8^+^ T cell counts, and drug regimens for all individuals are provided in Supplemental Figure 2. All participants had either acute or early HIV infection at the time of cohort entry. ART was initiated based on clinical guidelines at the time participants enrolled and their personal preferences.

### Serologic testing.

Presence of serum antibodies for HIV, EBV, HSV1, HSV2, and CMV infection were determined in the University of Washington Clinical Virology Lab, using plasma banked from the time closest to primary HIV infection.

### Screening of participants’ CD4^+^ T cells for peptide reactivity.

PBMCs were thawed and rested overnight at 37°C. Following incubation, CD8^+^ T cells were depleted according to the manufacturer’s instructions (EasySep Human CD8 Positive Selection Kit II, StemCell Technologies). CD8^+^ T cell–depleted PBMCs were plated in a 96-well round-bottom plate at 200,000 cells per well in a final volume of 250 μL of RPMI with 10% heat-inactivated human serum containing 10 ng/mL recombinant human IL-7 (Peprotech), 1 μM raltegravir (integrase inhibitor; https://www.beiresources.org/), and 15 nM efavirenz (non-nucleoside reverse transcriptase inhibitor; https://www.beiresources.org/). Peptide pools (Supplemental Table 3) were added to reach a final concentration of 2 μg/mL. Plates were incubated at 37°C. On days 3, 5, 7, and 10, half of the culture media was replaced with fresh RPMI/10% human sera containing a final concentration of 10 IU/mL IL-2 (Peprotech), 10 ng/mL IL-7, 1 μM raltegravir, and 15 nM efavirenz. On day 10, cells were restimulated with 2 μg/mL of specific peptide pools, Brefeldin A (MilliporeSigma), and GolgiStop (BD Biosciences) for intracellular cytokine staining. Staphylococcal enterotoxin B (MilliporeSigma) was used as a positive control at a concentration of 0.4 μg/mL and for compensation and fluorescence-minus-one (FMO) controls, with the latter used to distinguish positive and negative cell populations. Plates were incubated at 37°C for 6 hours and placed at 4°C until staining. Plates were washed twice with PBS prior to staining with Live Dead IR (Thermo Fisher Scientific). Cells were surface stained with optimized concentrations of anti-CD3 PECy7 (clone SK7), anti-CD8 BV421 (clone RPA T8), and anti-CD137 PE (clone 4B4-1) (all BD Biosciences) for 30 minutes. Cells were fixed and permeabilized according to the manufacturer’s instructions (Foxp3/Transcription Factor Fixation/Permeabilization, eBioscience) and stained with optimized concentrations of the following antibodies from BD Biosciences: anti–IFN-γ BV605 (clone B27), anti–TNF-α APC (clone 6401.1111), and anti–IL-2 BV711 (clone 5344.111) for 30 minutes prior to washing and fixing in PBS/1% paraformaldehyde (Electron Microscopy Sciences). For all flow cytometric analyses, fluorescence was measured using an LSRII (BD Biosciences) and all analyses were performed using FlowJo (Tree Star, Inc.).

### In vitro culturing of CD8^+^ T cell–depleted PBMCs.

PBMCs were thawed and rested overnight at 37°C. CD8^+^ T cell–depleted PBMCs were plated in a 24-well plate at 2 × 10^6^ cells per well in a final volume of 2 mL of RPMI supplemented with 10% heat-inactivated human serum containing 10 ng/mL recombinant human IL-7, 1 μM raltegravir, and 15 nM efavirenz. Peptide pools to which individuals were reactive (see Table 2) were added to reach a final concentration of 2 μg/mL and plates were incubated at 37°C. On days 3, 5, and 7, half of the culture media was replaced with fresh RPMI supplemented with 10% human sera containing a final concentration of 10 IU/mL IL-2, 10 ng/mL IL-7, 1 μM raltegravir, and 15 nM efavirenz. On day 10, cells were restimulated by addition of 2 μg/mL of peptide pools. Following a 30-hour incubation, cells were harvested and stained with Live Dead IR followed by optimized concentrations of the following antibodies from BD Biosciences: anti-CD3 PECy7 (clone SK7), anti-CD8 BV421 (clone RPA T8), and anti-CD137 PE (clone 4B4-1) for 30 minutes prior to washing. Live CD3^+^CD8^–^CD137^+^ cells were sorted on a BD Biosciences FACSAria cell sorter using FMO controls to set the sorting gates.

### VODA.

Standard curves for DNA quantitation were generated from HIV vector pNL4-3 (NIH AIDS Reagent Program) diluted from 10,000 copies/μL to 1 copy/μL at 1:10 serial dilution and 3 μL was used in a 20 μL qPCR assay. Human genomic DNA (Bioline) was diluted to 66.7 ng/μL to 0.0067 ng/μL at 1:10 serial dilution and 3 μL was used in a 20 μL qPCR assay. Both standards were added in the same wells in triplicate. DNA was aliquoted in 0.2 mL 8-strip tubes for single use and stored at –80°C for no more than 1 month. qPCR was performed in either 96-well or 384-well plates. PCR master mix consisted of 1× TaqMan Fast Advance Master Mix (Thermo Fisher Scientific), 140 nM of each HIV-1 probe (probeV1-LTR104-19, gag-B1, env-B2), 300 nM of each HIV-1 primer (NEC152, 5R633alt1, 5F1372alt1, 5R1504, 5F7724, and 5R7851), 120 nM human transferrin receptor probe (hTFR-exon-Cy5), 100 nM of each human primer (hTFR-exon-F and hTFR-exon-R2), 6 μL template or standards, and H_2_O to a total volume of 20 μL. H_2_O and 100 ng human genomic DNA were used as negative controls. PCR cycling parameters were as follows: initial denaturation at 95°C for 3 minutes, 45 cycles of 95°C for 5 seconds, 58°C for 15 seconds, and 60°C for 30 seconds on Quant Studio 6 (Thermo Fisher Scientific). Primers and probe for LTR, probeV1-LTR104-19, and NEC152 were published previously (55). Primers and probes for HIV env and gag and hTFR were designed using ABI Primer Express software; sequences are available in Supplemental Table 5.

### Cell proliferation measurements.

CD8^+^ T cell–depleted PBMCs were stained with 0.25 μL of CellTrace CFSE (Life Technologies) per 1 × 10^7^ cells in 1 mL PBS for 7 minutes and quenched with 2 mL FBS. After staining, PBMCs were plated in a 24-well plate at 2 × 10^6^ cells per well and stimulated with peptide pools at a concentration of 2 μg/mL in RPMI culture medium containing 1% penicillin-streptomycin, 10% human serum, 1 μM raltegravir, and 15 nM efavirenz. Anti-CD3/anti-CD28 Dynabeads, a human T cell activator, served as a positive control (Life Technologies). Stimulated cells were incubated for 5 days, after which cells were transferred to a 96-well round-bottom plate for staining. Live cells were identified by staining with the amine-reactive VIVID Pacific Blue viability marker (Life Technologies) for 20 minutes at room temperature. Following 2 washes in PBS, cells were stained with anti-CD3 PECy7 (BD Biosciences, clone SK7) and anti-CD8 PerCpCy5.5 (BD Biosciences, clone SK1). Fluorescence was measured using an LSRII (BD Biosciences) and all analyses were performed using FlowJo (Tree Star, Inc.)

### IS looping assay.

ISs were determined using an IS looping assay (ISLA), as previously described (16). In some cases, multiple displacement amplification (MDA) was performed prior to ISLA to amplify one HIV copy using HIV-specific primers (Supplemental Table 6).

### Sequence analyses.

Single genome sequences derived from ISLA or MDA-ISLA were edited using Geneious R8.1 (https://www.geneious.com/updates/geneious-prime-r8-1) to remove poor quality data, manually call ambiguous bases, and extract any mixed sequences (nucleotide sequences are available in the Supporting Data Values file). Subsequently, sequences were mapped to the human reference genome GRCh38.p2 with the IS pipeline developed in the Mullins Lab at the University of Washington (https://indra.mullins.microbiol.washington.edu/integrationsites/). The analysis pipeline utilized the final 40 bases of the HIV 3′ LTR to identify the site of provirus integration into the human genome. Sequences that mapped to an ambiguous location in the human genome due to HIV integration into a repetitive region were excluded. Gene names and genome locations were derived from Ensembl version 101 (http://aug2020.archive.ensembl.org/index.html) corresponding to GENCODE release 35 through annotations extracted from Ensembl’s BioMart data service. Genes were associated with ISs by computing the overlaps with IS locations. ISs falling within 10 kb upstream of a gene were considered within the promoter region for the gene. Unique ISs were determined by deduplicating on the tuple of (subject, chromosome/landmark, location, orientation). The multiplicity of an IS was defined as the number of times that exact (landmark, location, orientation) tuple is observed from independent amplification reactions within a participant. ISs with a multiplicity greater than 1 are assumed to originate from proliferating cells.

The location of ISs are reported in zero-origin, interbase coordinates; thus, location was identified between 2 nucleotides rather than a nucleotide (56). The top strand coordinate of the match was used as the location of integration. When the sequence matched the negative strand, the location of integration was defined as the value that is obtained by subtracting 4 from the top strand coordinate of the matched sequence, as previously described (57, 58). A total of 1,083 ISs from 7 participants in the ART-acute-HIV group and 632 ISs from 4 participants in the ART-chronic-HIV group was curated. After collapsing the ISs with the same location and orientation, we obtained 500 unique ISs in the ART-acute-HIV group and 520 unique ISs in the ART-chronic-HIV group. HIV-3′-HIV ISs are available in Retrovirus Integrations Database (https://rid.cancer.gov/bibliography.php). For our comparative analyses, we combined approximately 66,000 ISs from unstimulated primary CD4^+^ T cells infected with HIV-1_BaL_ for 48 hours (11) and approximately 3,000 primary resting CD4^+^ T cells infected with HIV NL4-3 for 96 hours (27) for a total of 69,184 unique in vitro ISs.

### Statistical analyses: IS comparisons.

All statistical analyses were conducted in R (https://www.R-project.org/). IS analyses were conducted following a prespecified tiered analysis plan. We curated gene sets from MSigDB v7.2 — H-“Hallmark” (50 gene sets), C2-“Canonical Pathways” (2,871 gene sets), and C2-“Chemical and genetic perturbations” (3,358 gene sets) — and filtered using the following criteria: (a) gene sets should contain at least 4 ISs from the in vivo data set, (b) ISs with assigned genes in the gene set must be from at least 2 unique participants, and (c) a priori minimum *P* value of 0.05 or less based on all possible permutations of ART-acute-HIV and ART-chronic-HIV labels among participants. Using these criteria, the number of gene sets within each of the 3 MSigDB collections was reduced to 22, 543, and 692 for H, C2.cp, and C2.cgp, respectively. We then used Fisher’s exact test to determine whether the number of unique ISs within genes and gene promoters in each gene set was independent of the source of the IS. Gene sets with significance at tier 1 (Holm-adjusted *P* ≤ 0.05) or tier 2 (FDR *q* ≤ 0.20 and unadjusted *P* ≤ 0.05) in either the ART-acute-HIV versus in vitro or ART-chronic-HIV versus in vitro analyses (Supplemental Table 4) were then considered for an in vivo–only analysis comparing ISs from participants in ART-acute-HIV and ART-acute-HIV groups. These gene sets are shown in Supplemental Table 4, along with the number of in vivo ISs overlapping or nonoverlapping with the genes in the gene set. Supplemental Table 4 also shows the odds ratio for ISs being associated with genes in the gene set, unadjusted Fisher’s exact test *P* values, Holm-adjusted *P* values, and FDR *q* values. For this in vivo–only analysis, we adjusted for multiple comparison only among the gene sets shown in Supplemental Table 4. This final test was not prespecified but will be prespecified in our future comparisons of IS analyses as co-primary with the unfiltered analysis that considers all gene sets. Limiting the comparison of in vivo gene sets already known to differ in one or both of the in vivo comparison groups versus the in vitro data improves power for identifying relevant differences in vivo by a priori considering only those gene sets with evidence of some enrichment or depletion compared to what we expect from the in vitro integration experiments.

### Statistics: VODA.

The estimates of HIV copies per 10^6^ cells depicted in Figure 1 are based on logs of ratios of estimated numbers of HIV copies to estimated number of cells (using the hTFR housekeeping gene), averaged over 2 replicates. In some cases, only one replicate exceeded the limit of detection (see below), and in these cases the estimate is based on this replicate only (indicated as open circles). When neither replicate exceeded the limit of detection, the value is shown as an open square at 1.

We leverage the independence of the replicates and of the measurement error in PCR across reactions to obtain a pooled variance estimate for the uncertainty in these estimators. Error bars are used to indicate 2 standard errors, estimated using the following procedure. First, we estimate variances of the normally distributed error of the difference of log concentration estimates for numerator (HIV LTR) and denominator (hTFR) for each replicate separately; using each fitted standard curve (for predicting log concentration from C_T_), one for HIV LTR and the other for hTFR, we estimate the variance of the mean of the 2 replicates by considering the variance of each replicate as the sum of the estimated residual variance from the fitted standard curve simple linear regression models for the corresponding plate. The final standard error of the mean is computed as one-half the square root of the sum of the variance estimates for each replicate. A complete set of such curves is given in Supplemental Figure 4. These curves give a normally distributed prediction of log concentration for any given observed C_T_ value. Since the difference of these independent log concentration estimates (for HIV LTR and for hTFR) is approximately normally distributed with variance given by the sum of the variances of each component, this formula yields the estimated error in the log ratio.

The effective limit of detection varied across these results. To aid in interpretation of these estimated values and confidence limits, we have included dotted lines on each panel in Supplemental Figure 4 to indicate the value of copies per million cells that would be estimated if you observed a C_T_ value corresponding to 1 viral copy in the reaction. Sometimes C_T_ values beyond the threshold of 1 copy may be considered reliable; however, these lines can be considered effective limits of detection in that values below this line are as reliable as C_T_ values beyond that threshold. Specifically, we compute these values as 10^6^ per the geometric mean of the log hTFR copy values (over the 1–2 available replicate values).

### Data availability.

Data used to derive Figures 1–3 are included as a Supporting Data Values file.

### Study approval.

Specimens from participants were obtained after written informed consent was given, following a protocol approved by the University of Washington’s Human Subjects’ Institutional Review Board.

## Author contributions

LMF and HH designed these studies. JM, ACC, and JIM enrolled participants into the study cohort and provided clinical specimens. AG, JJ, LC, BK, SS, DK, and SM conducted the experiments. JJ, AG, DS, EK, PTE, JIM, and LMF analyzed and interpreted the data. DS, EK, and PTE performed statistical data analyses. JJ, AG, and LMF wrote the manuscript. All authors reviewed and contributed revisions to the manuscript. JJ and AG were deemed co–first authors, as JJ performed molecular biology and virology experiments and led drafting of the manuscript and AG led immunology experiments.

## Supplementary Material

Supplemental data

Supporting data values

## Figures and Tables

**Table 2:**
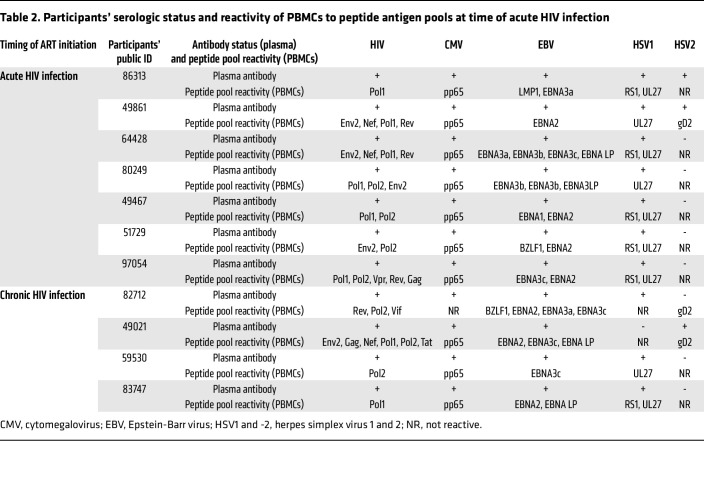
Participants’ serologic status and reactivity of PBMCs to peptide antigen pools at time of acute HIV infection

**Table 1:**
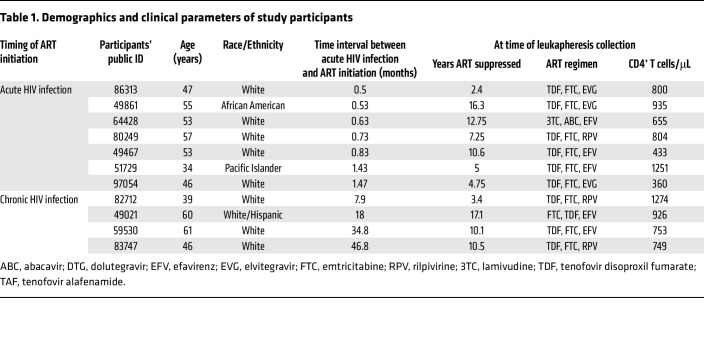
Demographics and clinical parameters of study participants

## Version 1 06/04/2024

In-Press Preview

## Version 2 07/15/2024

Electronic publication
