# Supplementary material for: Antigen specificities and proviral integration sites differ in HIV-infected cells by timing of antiretroviral treatment initiation
Source: J Clin Invest. 2024 Jun 4;134(14):e159569. doi: 10.1172/JCI159569 (PMC11245156; doi:10.1172/JCI159569)
Supplement: Supplemental data [file jci-134-159569-s178.pdf]

Supplementary Table 1. Cell numbers before and after *in vitro* peptide antigen expansion

PID	Peptide Antigen Pool	Day 0 CD8 depleted PBMCs (x10^6)	Day 0 CD137$^+$ % of total PBMCs	Day 0 CD4$^+$CD137$^+$ cells (x10^6)	Day 11 Pre-sort CD4$^+$CD137$^+$ cells (x10^6)	Day 11 Post-sort CD4$^+$CD137$^+$ cells (x10^6)
86313	HIV	34	0.002	0.07	3.14	1.62
86313	EBV	36	0.008	0.38	4.86	3.5
86313	HSV1	36	0.005	0.17	9.90	8.31
49861	CMV	30.8	0.004	0.12	13.17	4.16
49861	HSV2	66	0.035	2.29	12.91	8.13
49861	HSV1	33.8	0.037	1.24	5.69	2.87
49861	EBV	35.76	0.028	1.00	5.97	2.65
49861	HIV	33.8	0.034	1.16	13.94	6
64428	CMV	28	0.011	0.31	7.03	3.24
64428	HIV	22	0.045	1.01	2.23	0.87
64428	HSV1	24	0.052	1.24	2.16	0.68
64428	EBV	38	0.044	1.70	4.94	0.79
80249	CMV	30	0.008	0.24	2.95	2.99
80249	EBV	36	0.013	0.47	3.87	2.43
80249	HIV	34	0.011	0.39	4.26	3.88
80249	HSV1	29	0.005	0.14	2.34	1.09
49467	CMV	35.7	0.046	1.64	3.81	1.38
49467	EBV	26	0.021	0.54	2.46	2.09
49467	HSV1	26	0.022	0.57	4.26	2.64
49467	HIV	27	0.032	0.81	3.17	2.69
51729	CMV	30	0.006	0.19	5.81	4.66
51729	HIV	28	0.008	0.23	3.04	1.92
51729	HSV1	28	0.013	0.35	7.10	5.51
51729	EBV	30	0.007	0.21	1.56	1.95
97054	CMV	35	0.015	0.51	5.41	4.9
97054	EBV	32	0.013	0.43	2.90	2.27
97054	HSV1	32	0.013	0.40	5.17	4.6
97054	HIV	34	0.006	0.22	2.96	1.6
82712	HSV2	26	0.075	1.95	3.32	1.45
82712	HIV	28	0.007	1.49	2.43	1.74
49021	CMV	40	0.010	0.39	10.44	5.1
49021	HSV2	40	0.023	0.93	12.61	8
49021	HIV	48	0.012	0.56	9.19	3.34
49021	EBV	36	0.010	0.72	5.67	4.07
59530	CMV	34	0.025	0.85	6.69	5.58
59530	HSV1	39	0.025	0.99	7.66	4.77
59530	EBV	36	0.023	0.84	4.57	2.7
59530	HIV	36	0.032	1.15	5.90	3.56
83747	CMV	42	0.015	0.63	11.80	6.88
83747	HIV	44	0.029	1.29	3.10	0.97
83747	EBV	60	0.019	1.22	6.72	1.38
83747	HSV1	42	0.033	1.40	8.02	4.74

Supplementary Table 2. HIV DNA in $CD3^+CD8^-CD137^+$ and $CD3^+CD8^-CD137^-$ cells after *in vitro* peptide antigen expansion

		HIV LTR copies per 10^6 cells	
PID	Peptide Antigen Pool	$CD3^+CD8^-CD137^+$	$CD3^+CD8^-CD137^-$
86313	EBV	Undetectable	17.06
	HIV	439.17	109.21
	HSV1	1.32	42.49
49861	CMV	15.27	42.62
	EBV	128.35	64.33
	HIV	763.36	248.35
	HSV-1	104.26	94.47
	HSV-2	130.47	76.82
64428	CMV	692.64	218.21
	EBV	331.66	307.13
	HIV	3799.63	734.70
	HSV-1	398.51	329.41
80249	CMV	44.03	92.65
	EBV	30.70	164.56
	HIV	4835.84	2515.71
	HSV1	161.73	71.77
49467	CMV	434.58	592.82
	EBV	920.64	1758.03
	HIV	8777.26	2185.93
	HSV-1	549.37	952.13
51729	CMV	145.58	209.49
	EBV	192.66	178.75
	HIV	5662.89	1554.99
	HSV1	Undetectable	54.57
97054	CMV	17663.25	7037.19
	EBV	1497.41	1980.50
	HIV	16154.83	4615.42
	HSV1	1693.47	2992.47
82712	HIV	18791.64	8680.44
	HSV2	4697.68	6495.51
49021	CMV	840.74	475.00
	EBV	239.00	479.34
	HIV	8966.64	1073.61
	HSV-2	223.73	416.00
59530	CMV	3421.79	2532.35
	EBV	6251.76	2828.65
	HIV	7704.99	3138.96
	HSV1	1432.80	1009.42
83747	CMV	9804.19	6950.97
	EBV	8572.61	16597.91
	HIV	8178.71	7706.38
	HSV-1	8992.38	6379.12

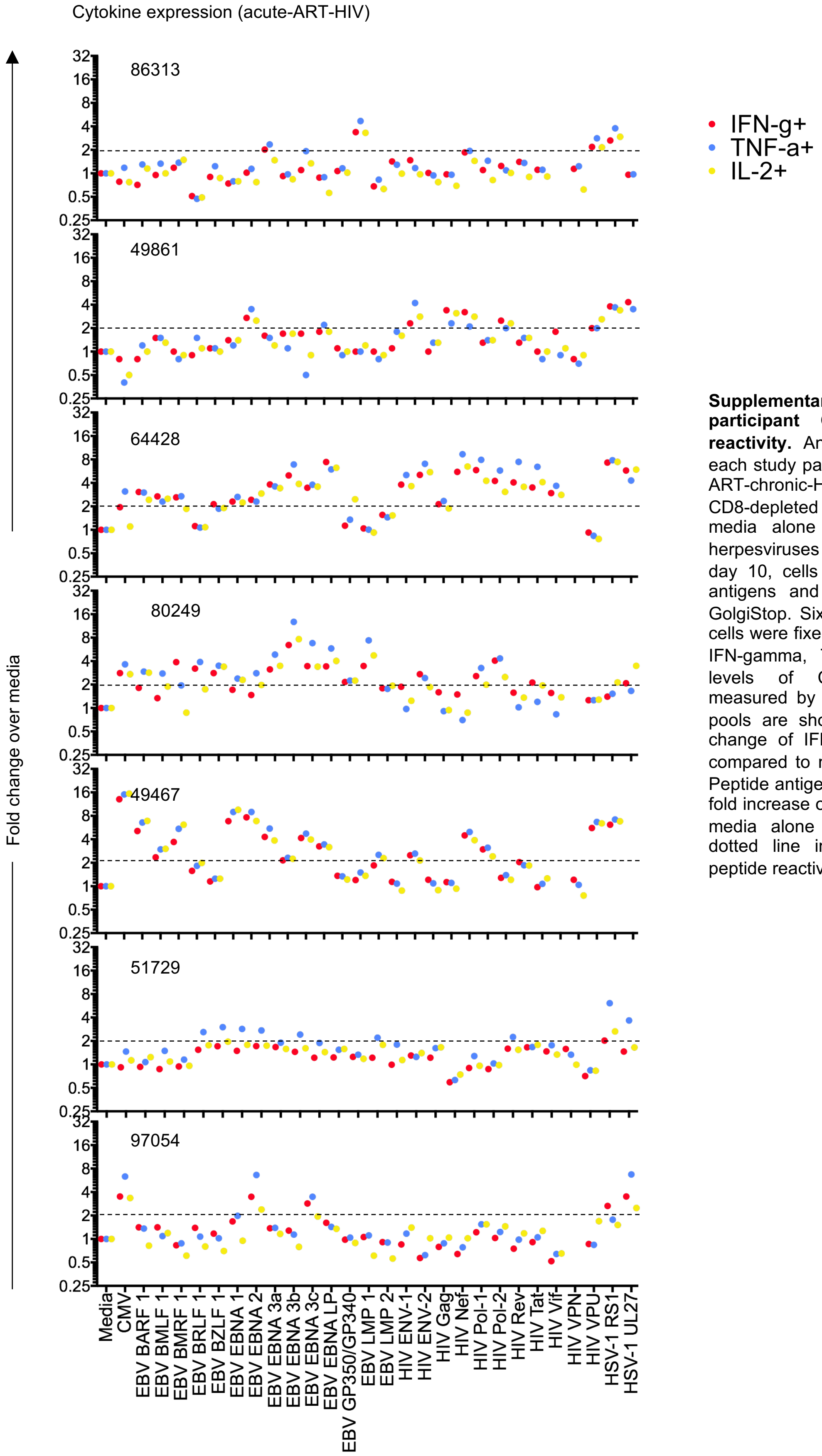

Supplementary Figure 1. Screening of participant CD4⁺ T cells for peptide reactivity. Antigen-specific CD4⁺ T cells for each study participant in the ART-acute-HIV or ART-chronic-HIV groups were assessed using CD8-depleted PBMCs incubated at 37˚C with media alone (negative control) or HIV or herpesviruses peptide antigens for 10 days. On day 10, cells were restimulated with peptide antigens and treated with Brefeldin A and GolgiStop. Six hours after incubation at 37˚C, cells were fixed, stained for CD3, CD8, CD137, IFN-gamma, TNF-alpha, and IL-2. Cytokine levels of CD3⁺CD8⁻CD137⁺ cells were measured by flow cytometry. Peptide antigen pools are shown along the x-axis and fold-change of IFN-gamma, TNF-alpha, and IL-2 compared to media alone is shown on y-axis. Peptide antigens that resulted in at least a two-fold increase of all three cytokines compared to media alone were deemed reactive. Black dotted line indicates two-fold threshold for peptide reactivity.

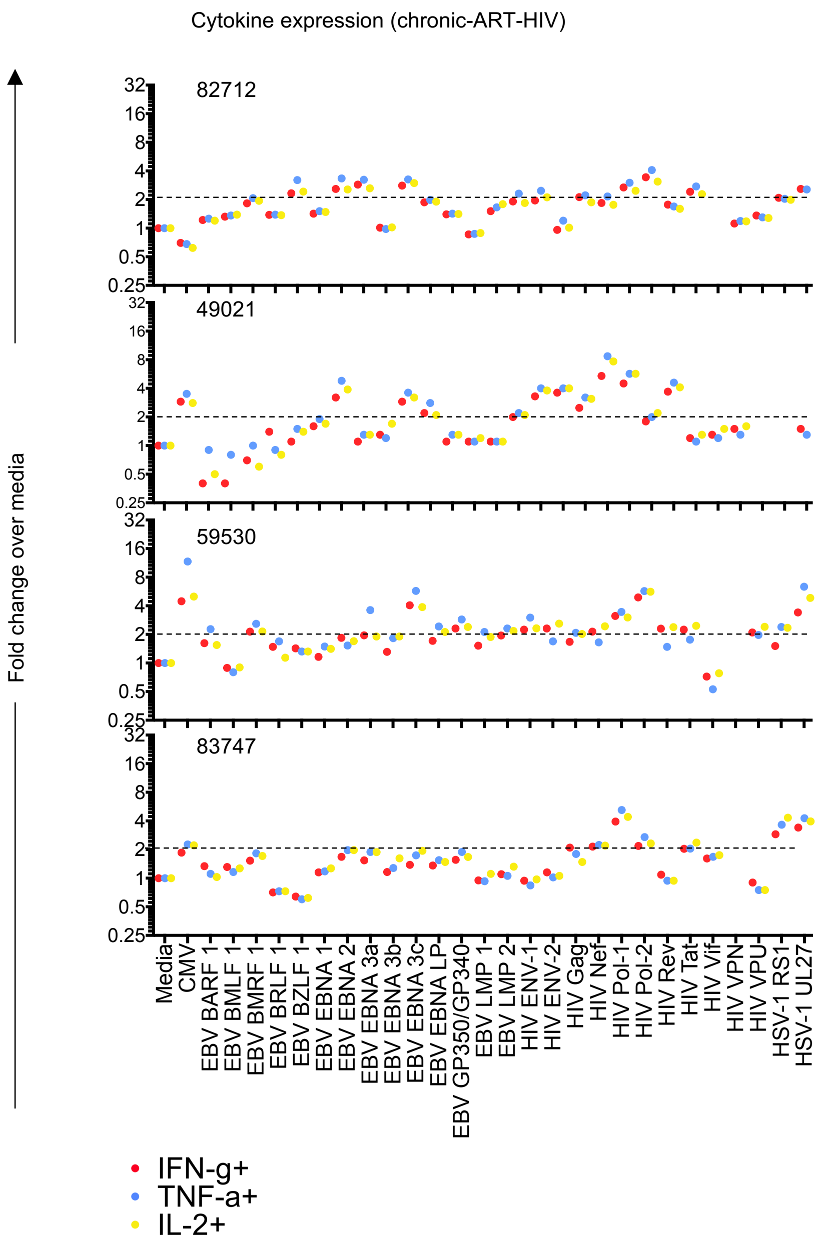

Supplementary Figure 1 (con't). Screening of participant CD4⁺ T cells for peptide reactivity. Antigen-specific CD4⁺ T cells for each study participant in the ART-acute-HIV or ART-chronic-HIV groups were assessed using CD8-depleted PBMCs incubated at 37˚C with media alone (negative control) or HIV or herpesviruses peptide antigens for 10 days. On day 10, cells were restimulated with peptide antigens and treated with Brefeldin A and GolgiStop. Six hours after incubation at 37˚C, cells were fixed, stained for CD3, CD8, CD137, IFN-gamma, TNF-alpha, and IL-2. Cytokine levels of $CD3^{+}CD8^{-}CD137^{+}$ cells were measured by flow cytometry. Peptide antigen pools are shown along the x-axis and fold-change of IFN-gamma, TNF-alpha, and IL-2 compared to media alone is shown on y-axis. Peptide antigens that resulted in at least a two-fold increase of all three cytokines compared to media alone were deemed reactive. Black dotted line indicates two-fold threshold for peptide reactivity.

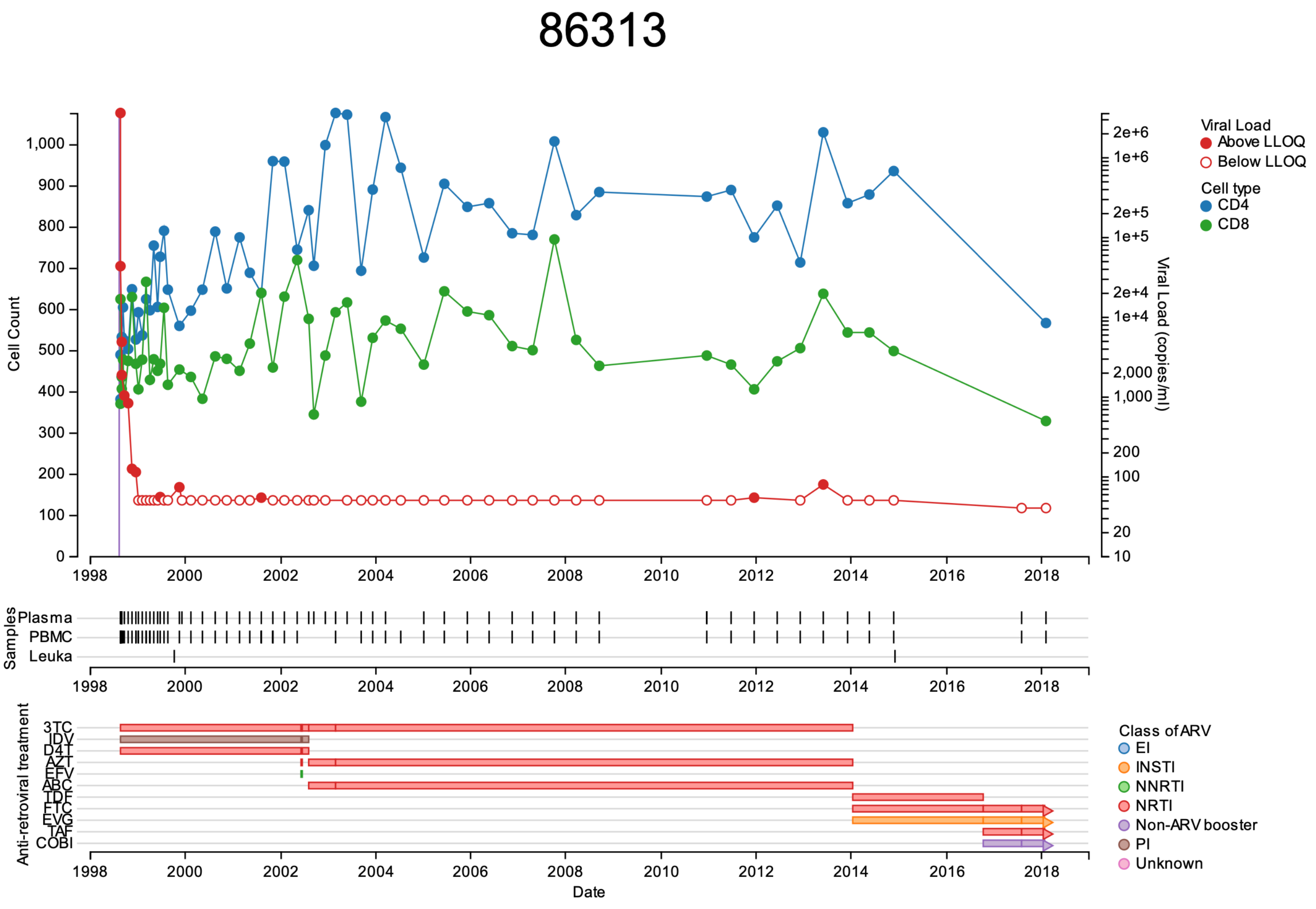

Supplementary Figure 2. Participants' plasma HIV RNA loads, CD4+ and CD8+ T cell counts (#/uL), and ART regimens since the time of HIV acquisition. Plasma HIV RNA values (red), CD4+ (blue), and CD8+ (green) T-cell counts, and antiretroviral treatments (below x-axis) are shown for each participant. Plasma HIV RNA symbols are filled when HIV RNA was detected and are open circles when below the lower-limit-of-quantification (either 40 or 50c/mL, depending on clinical assay employed). Antiretroviral treatments and time intervals prescribed are shown by horizontal bars. EI: entry inhibitor, INSTI: integrase strand transfer inhibitor, NNRTI: non-nucleoside reverse transcriptase inhibitor, NRTI: nucleoside reverse transcriptase inhibitor, PI: protease inhibitor.

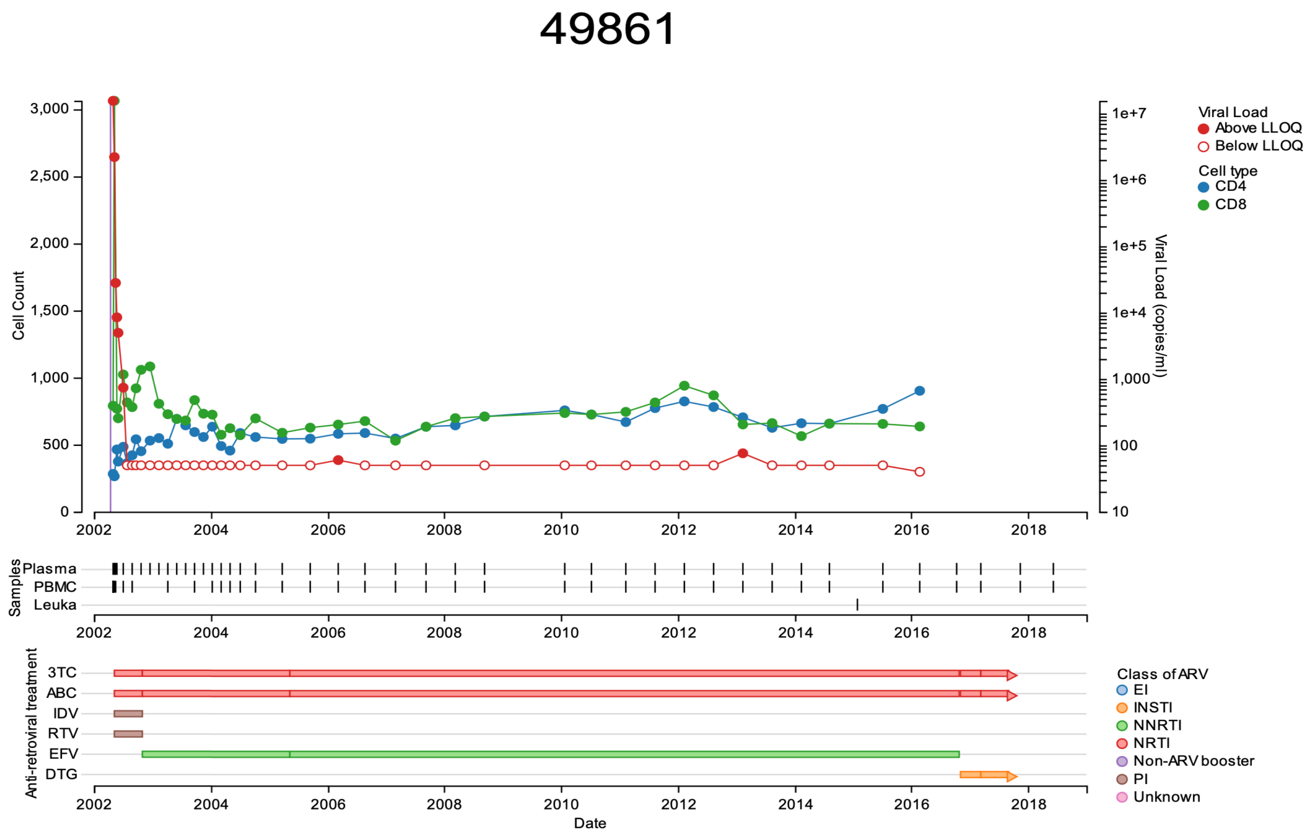

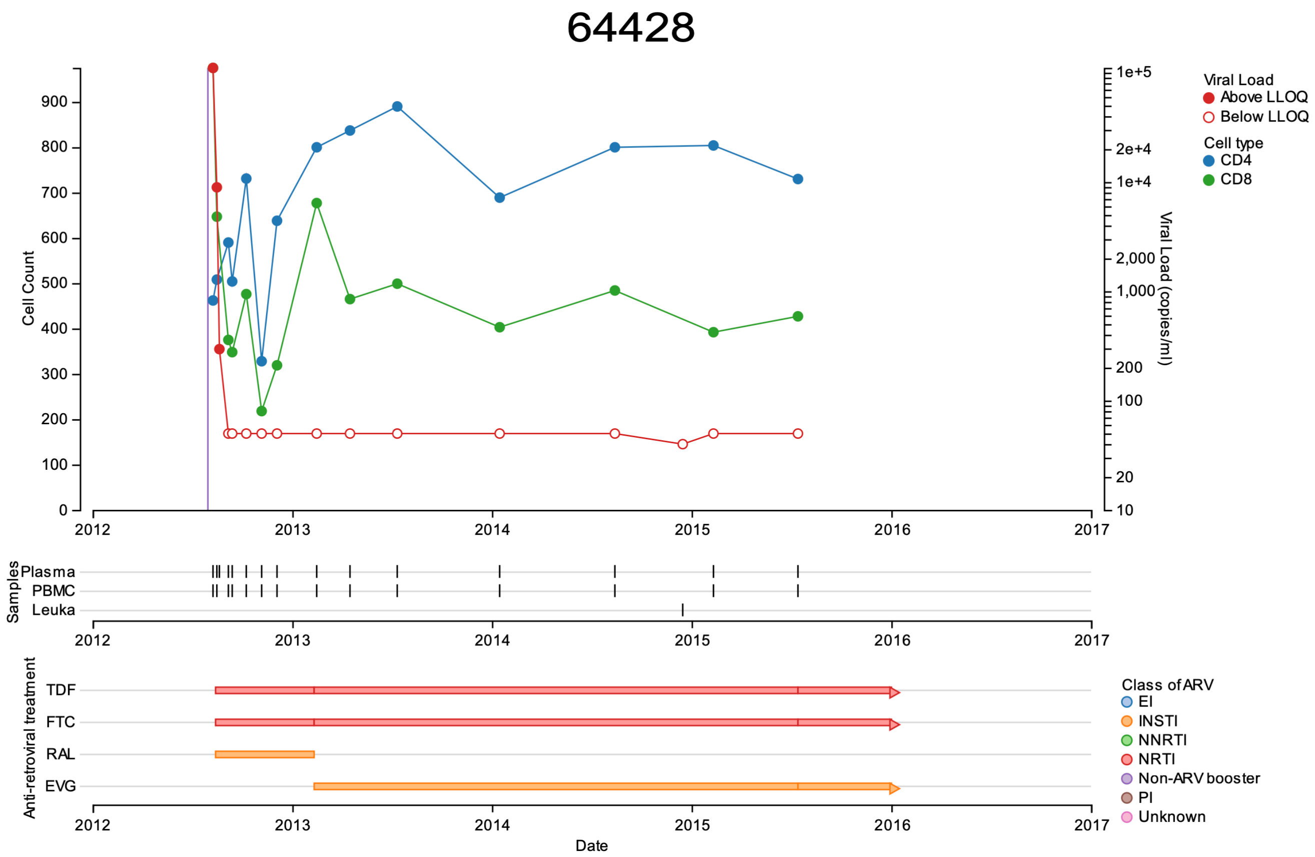

Supplementary Figure 2. (con't)

80249

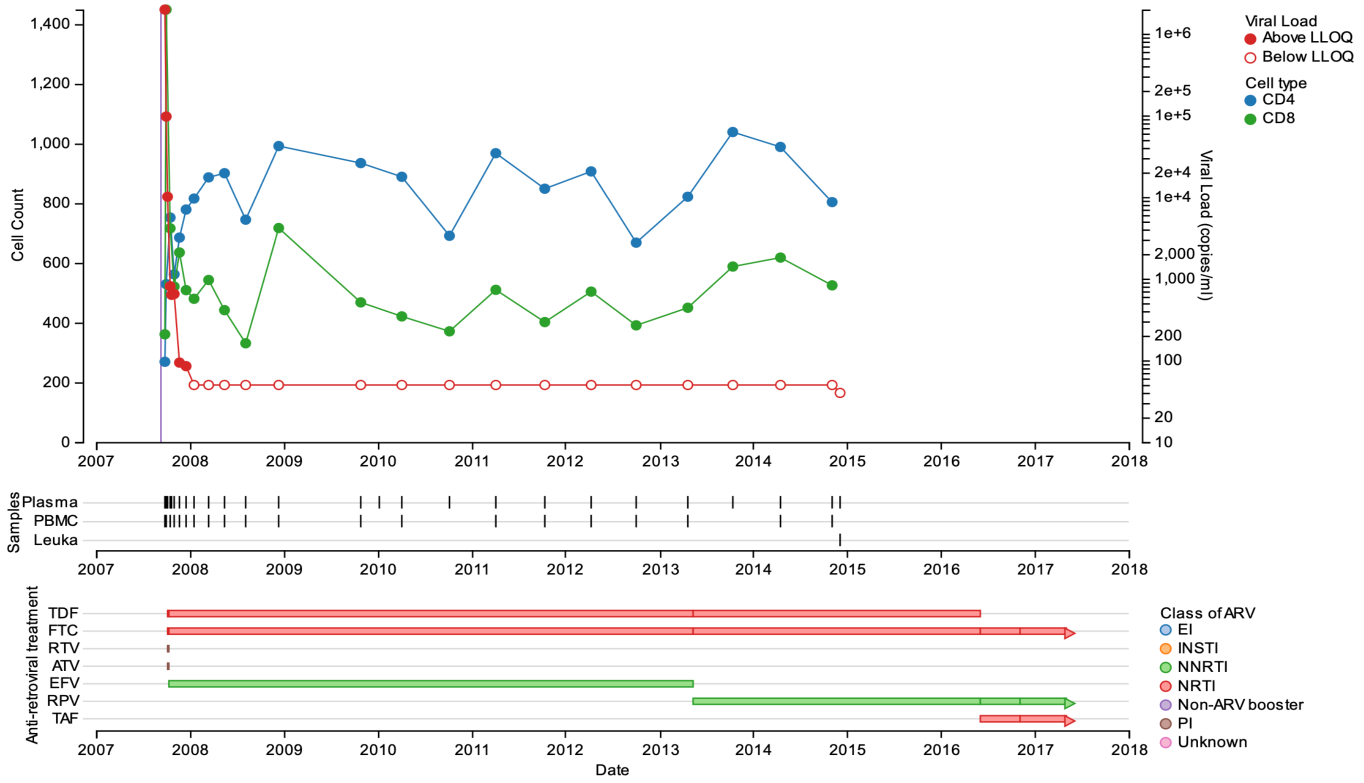

49467

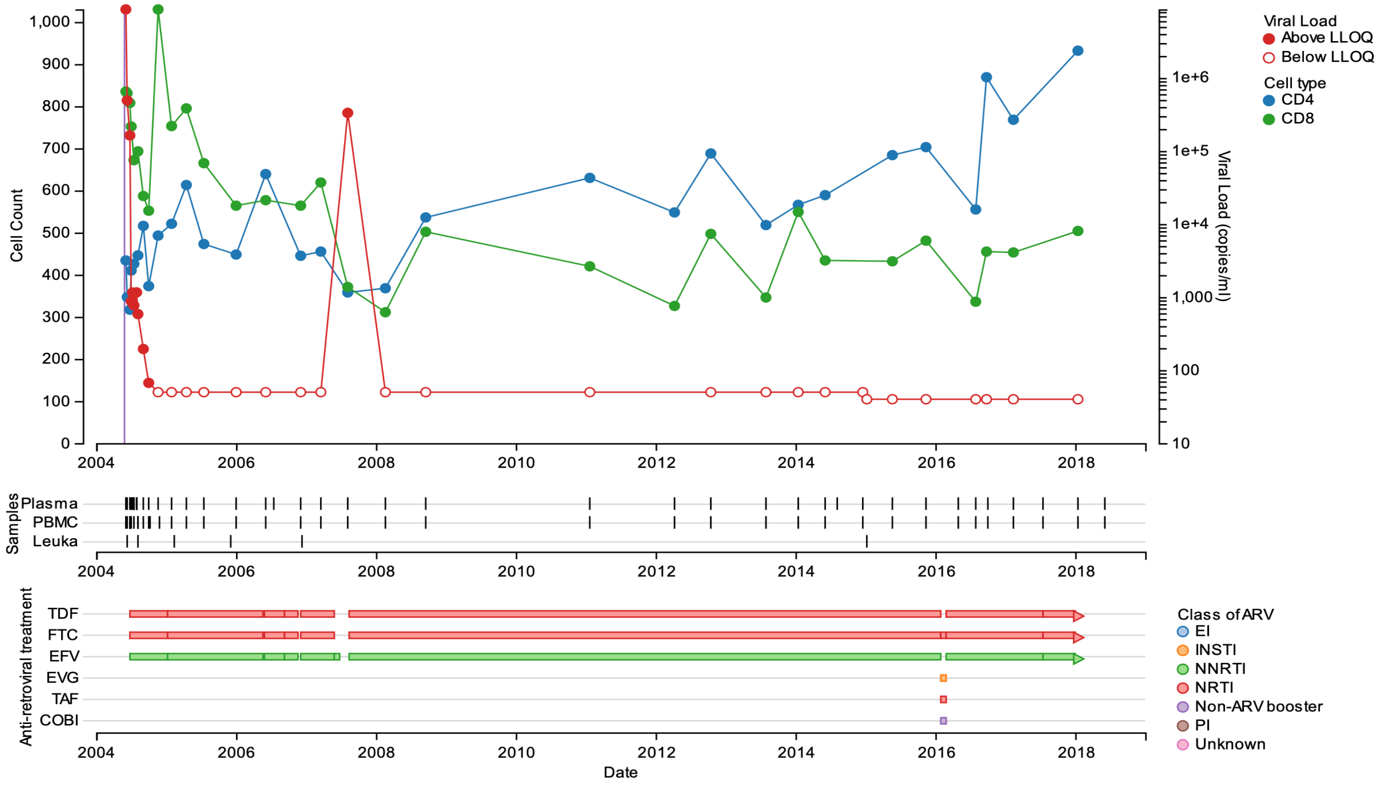

Supplementary Figure 2. (con't)

51729

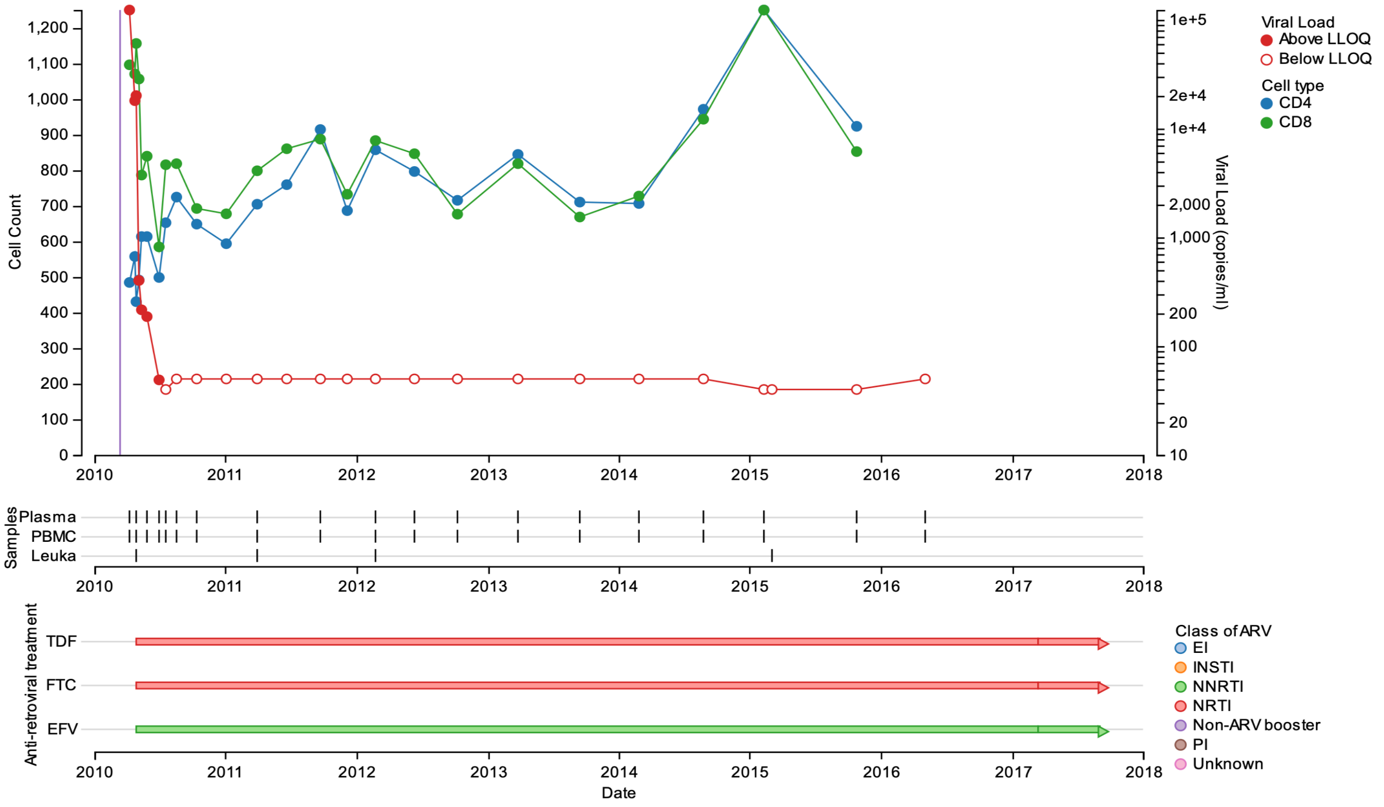

83747

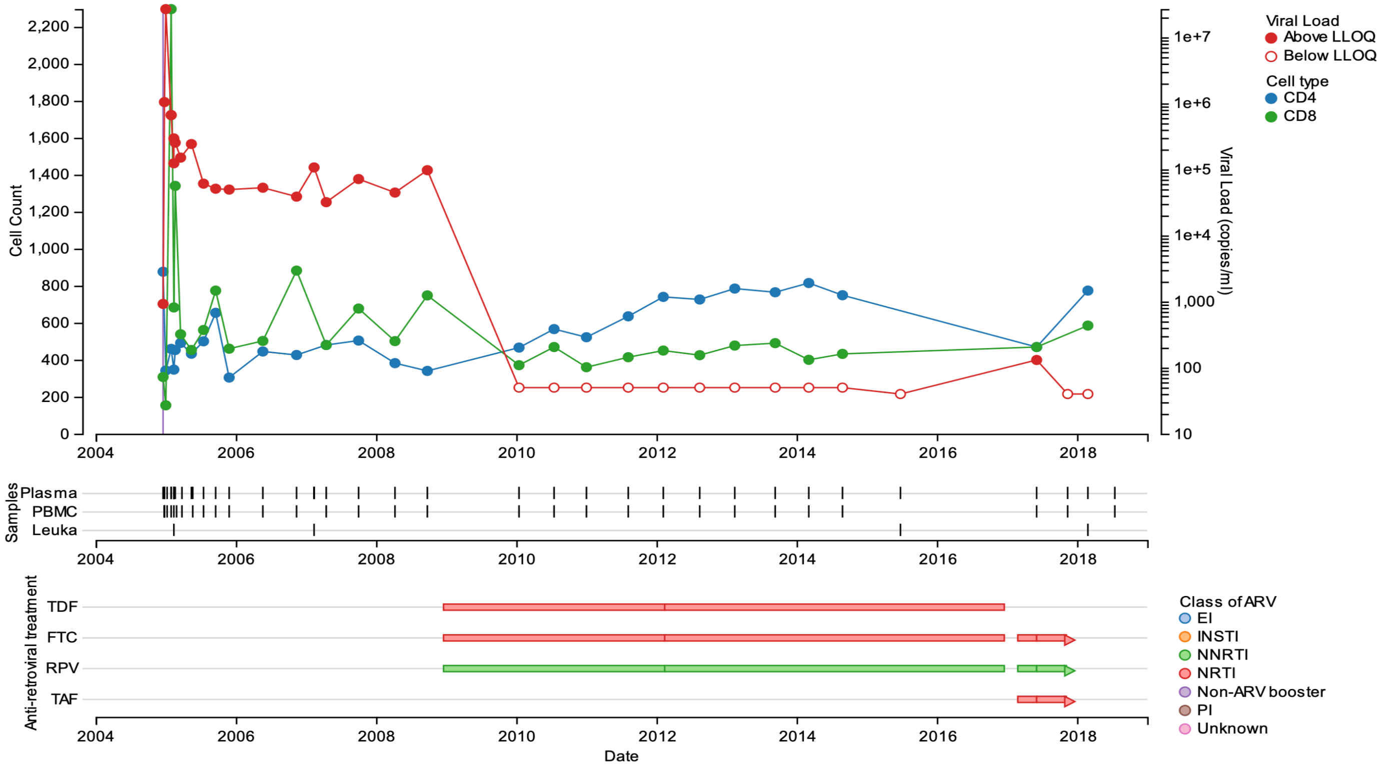

Supplementary Figure 2. (con't)

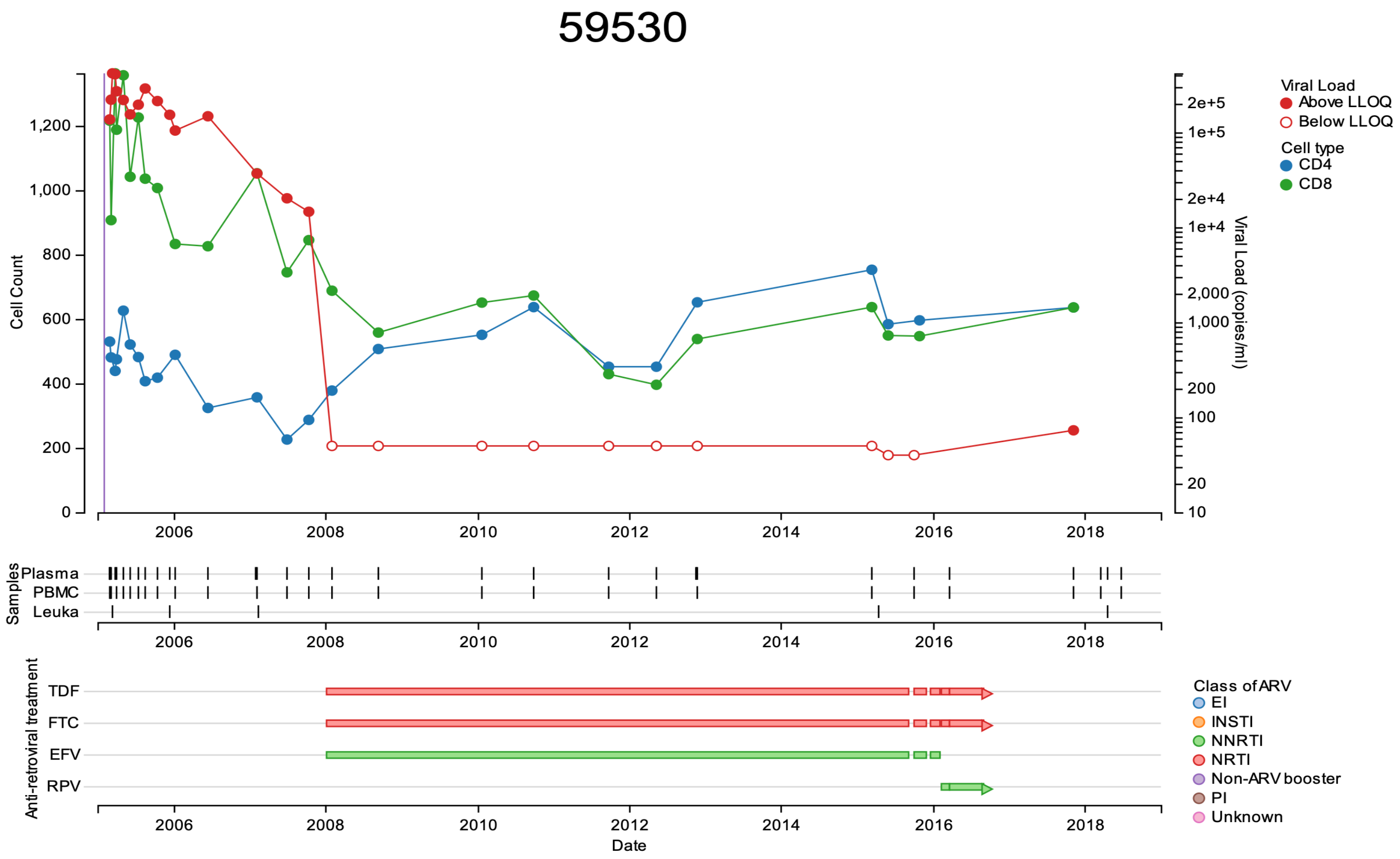

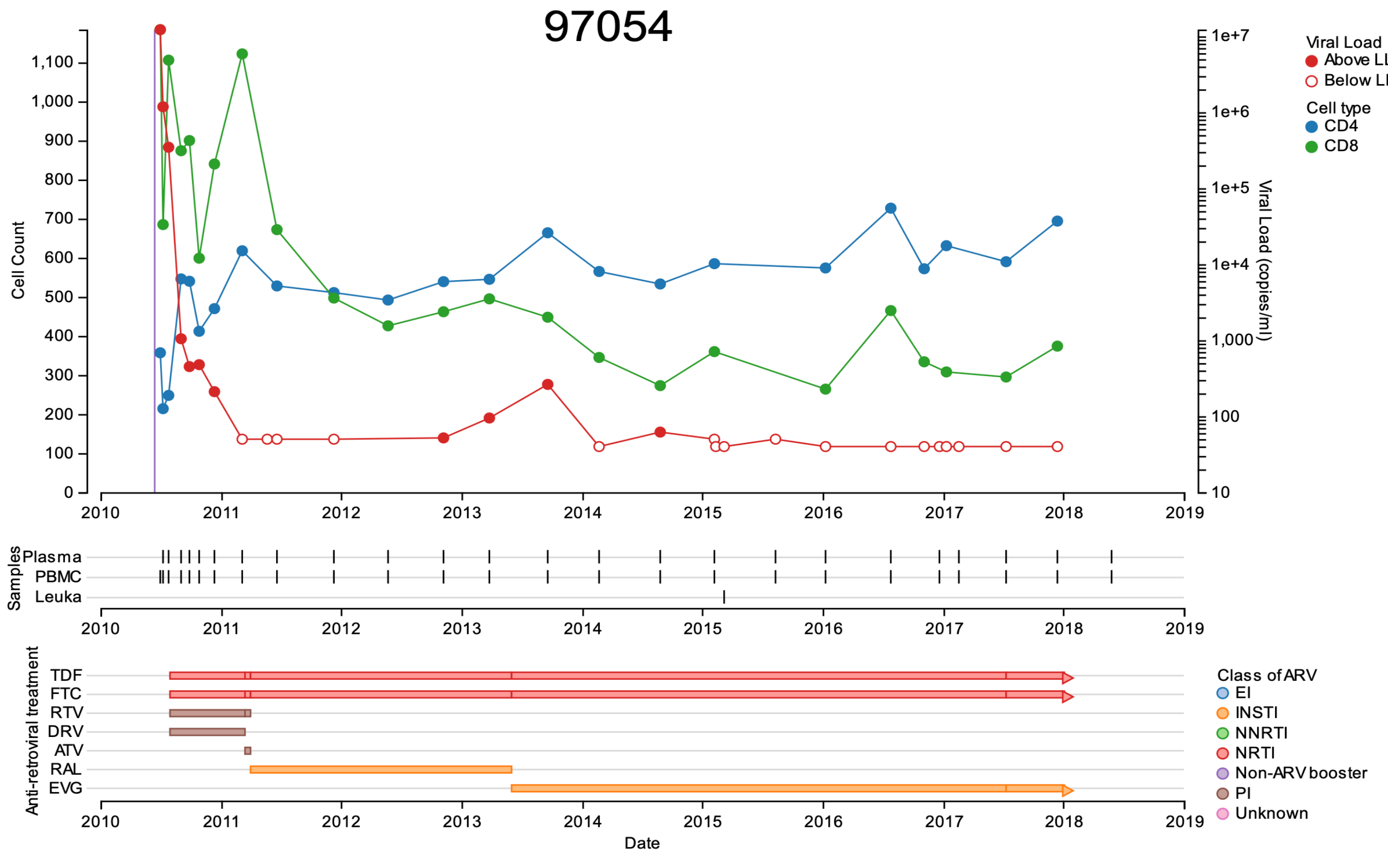

Supplementary Figure 2. (con't)

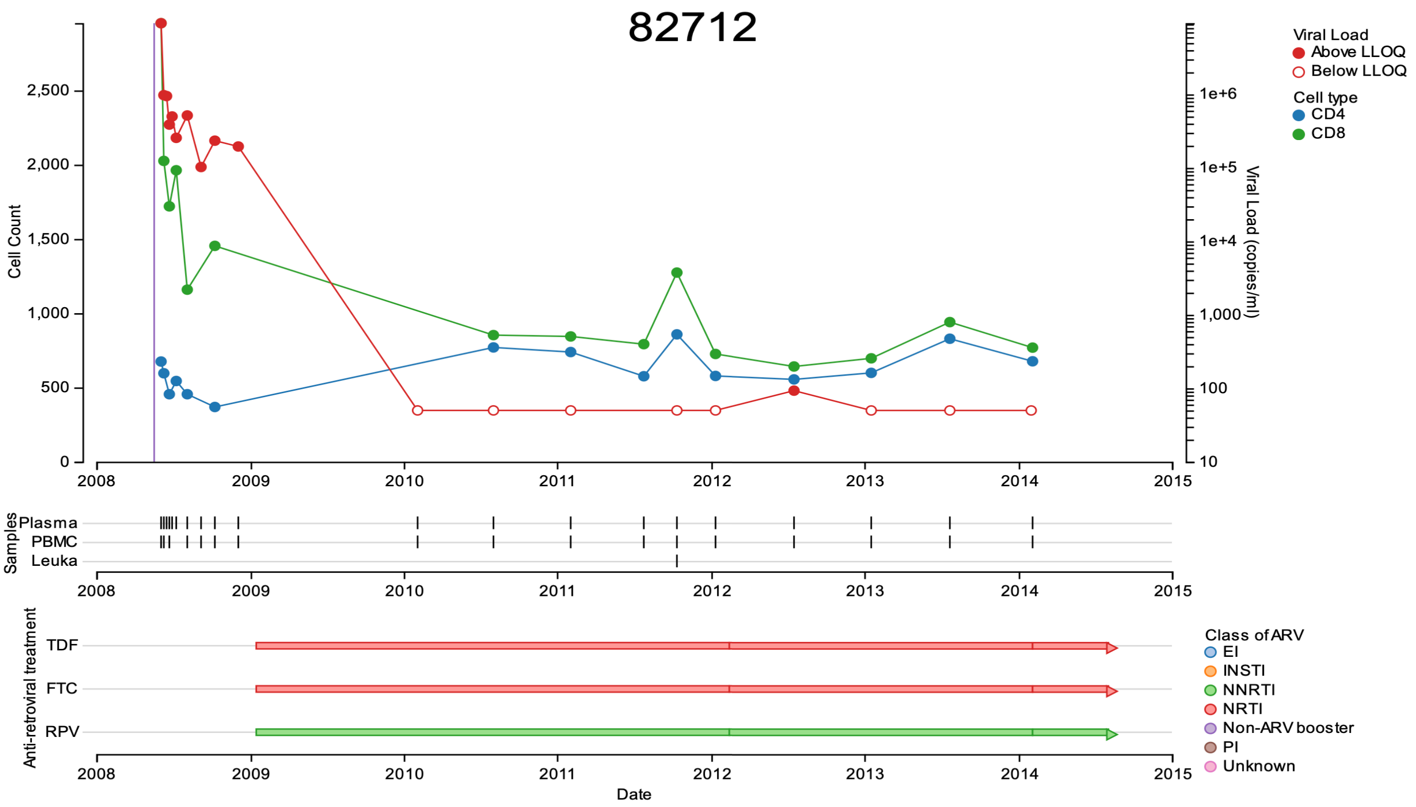

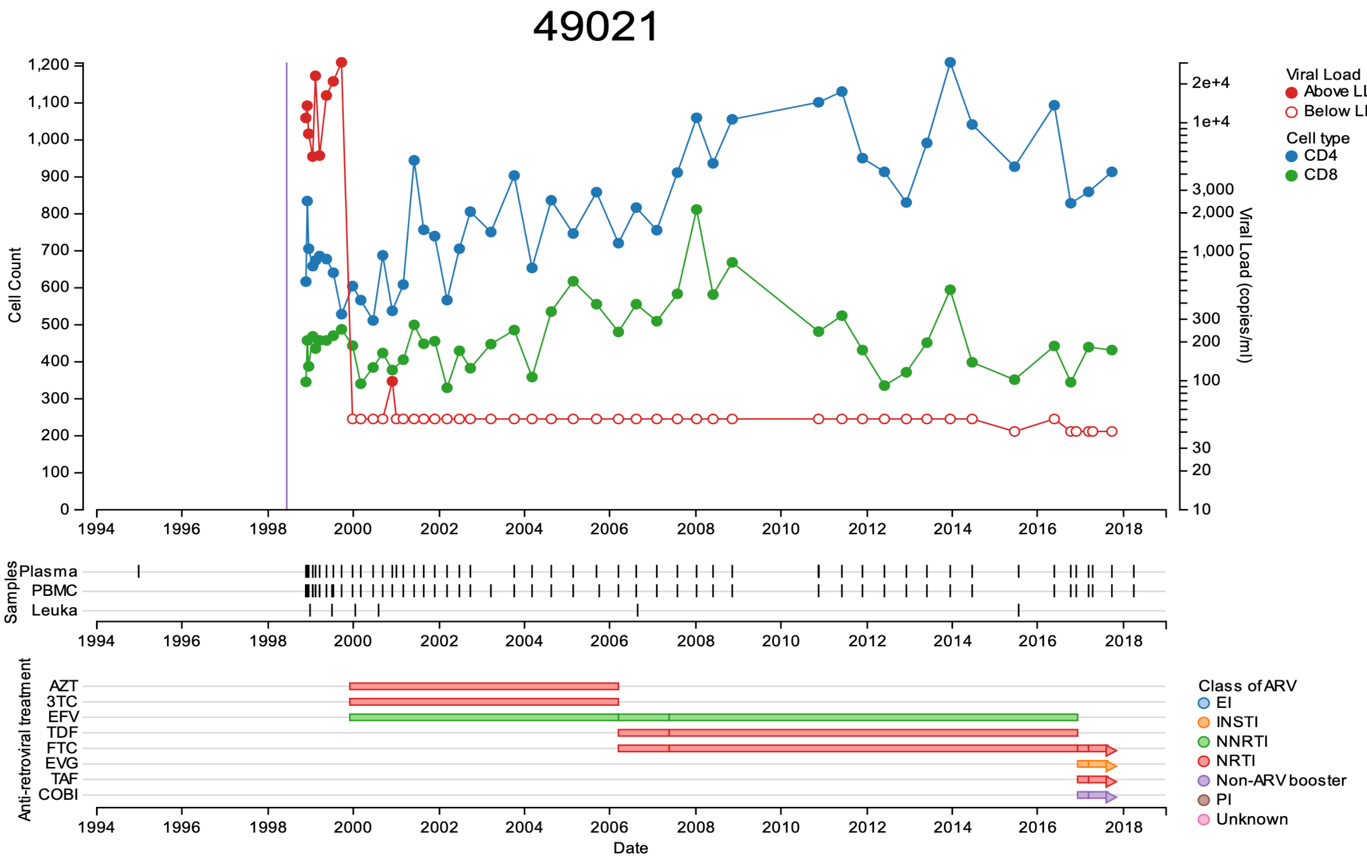

Supplementary Figure 2. (con't)

A. Proliferation of CD3+CD8-CD137+ cells after antigen stimulation (acute-ART-HIV)

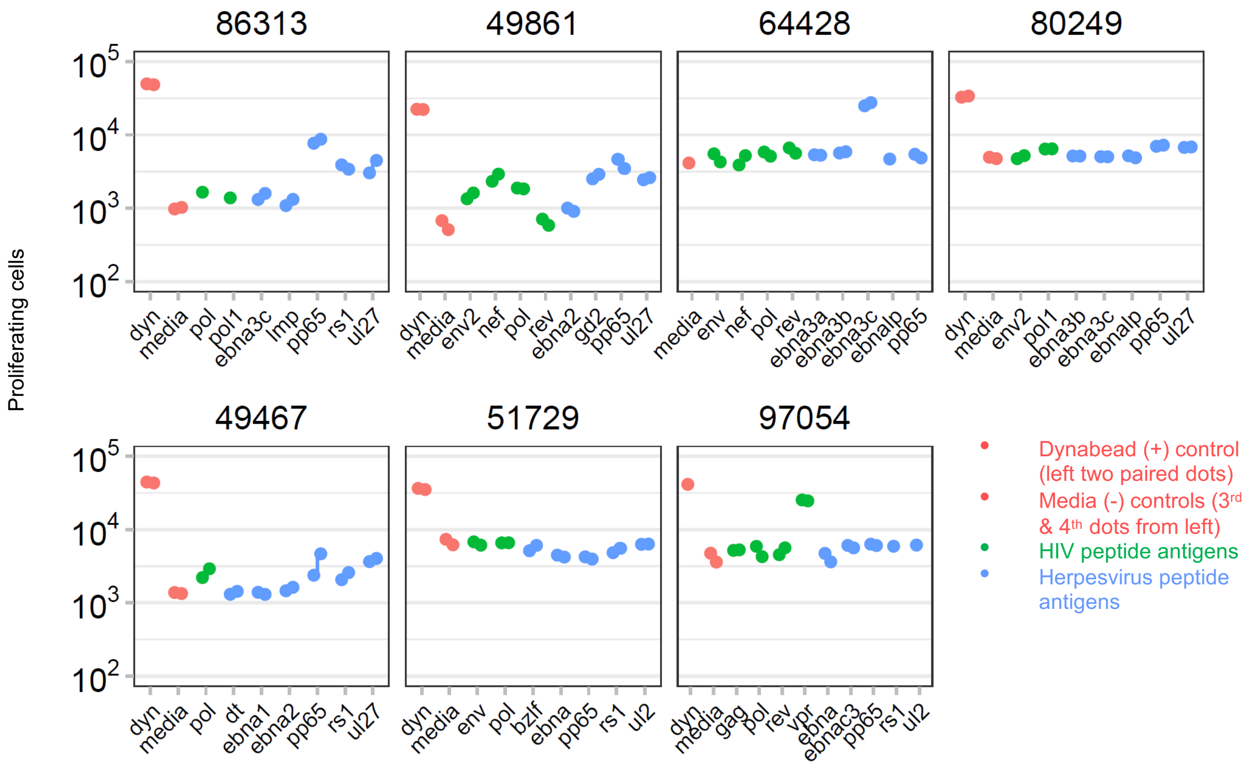

Supplementary Figure 3. Proliferation of antigen-specific CD4⁺ T cells after *in vitro* peptide antigen stimulation. To measure relative proliferation of CD4⁺ T cells after peptide antigen stimulation, $2x10^6$ CD8-depleted PBMCs were stained with CFSE and stimulated with peptide antigens. Five days post stimulation, the number of proliferating cells were quantified by flow cytometry. Stimulation with anti-CD3/CD28 Dynabeads (Dyn) (Life Technologies AS, Norway) were used as a positive control and tissue culture media in the absence of peptide antigens (media) served as a negative control. Peptide antigen pools used for each participant from the ART-acute-HIV (**Panel A**) and ART-chronic-HIV (**Panel B – next page**) groups were based on the individual's peptide reactivity (see **Supplementary Figure 1**). Numbers of proliferating cells were determined based on CFSE dilution using FlowJo and are reported in graphs. Red dots indicate controls, green indicates HIV peptide antigens, and blue indicates herpesviruses peptide antigens. Experiments were done in duplicates. Intra-individual differences between the highest number of proliferating cells in response to HIV peptide antigen stimulation minus the highest number of proliferating cells in response to herpesviruses peptide antigen stimulation are shown (**Panel C – next page**). The median value is below zero, resulting in a one-sided Wilcoxon p-value of 0.958, indicating that cells do not proliferate more in response to HIV stimulation.

EBV antigens: barf1, bmlf1, bmrf1, brlf1, bzlf1, ebnalp, ebna1, ebna2, ebna3a, ebna3b, ebna3c, lmp1, lmp2. HIV antigens: vpu, rev, vpr, pol1, pol2, pol, gag, vif, env1, env2, tat, nef. HSV-1 antigens: rs-1, ul27. HSV2 antigen: gD2. CMV antigen: pp65.

B. Proliferation of CD3+CD8-CD137+ cells after antigen stimulation (chronic-ART-HIV)

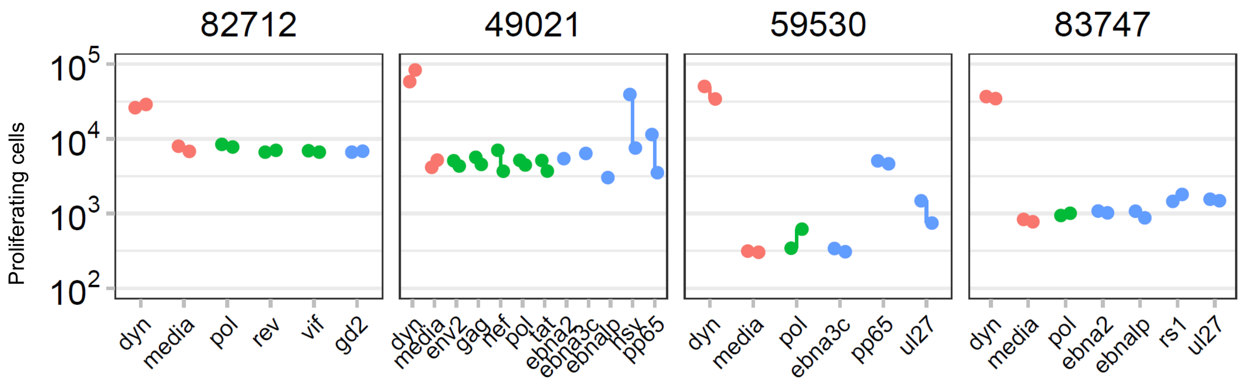

- Dynabead (+) control (left two paired dots)
- Media (-) controls (3rd & 4th dots from left)
- HIV peptide antigens
- Herpesvirus peptide antigens

C. Intra-individual differences in peptide antigen-induced proliferation

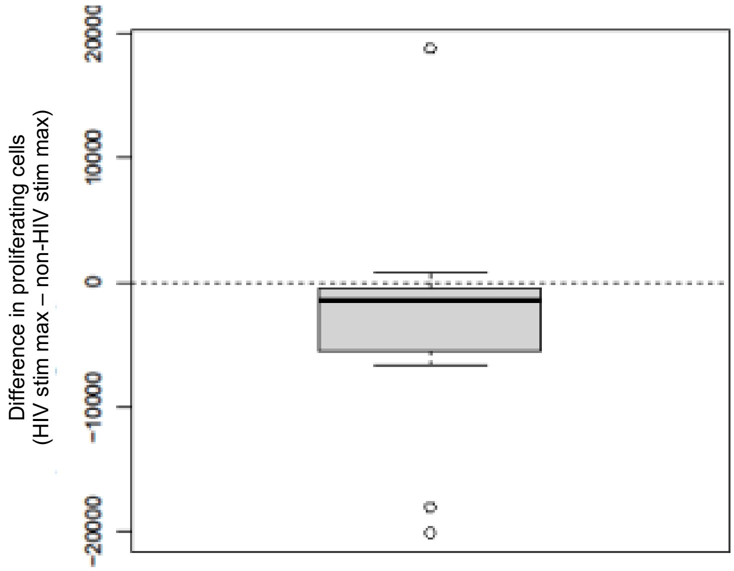

Supplementary Figure 3 con't. Proliferation of antigen-specific CD4+ T cells after *in vitro* peptide antigen stimulation. To measure relative proliferation of CD4+ T cells after peptide antigen stimulation, $2x10^6$ CD8-depleted PBMCs were stained with CFSE and stimulated with peptide antigens. Five days post stimulation, the number of proliferating cells were quantified by flow cytometry. Stimulation with anti-CD3/CD28 Dynabeads (Dyn) (Life Technologies AS, Norway) were used as a positive control and tissue culture media in the absence of peptide antigens (media) served as a negative control. Peptide antigen pools used for each participant from the ART-acute-HIV (**Panel A – previous page**) and ART-chronic-HIV (**Panel B**) groups were based on the individual's peptide reactivity (see **Supplementary Figure 1**). Numbers of proliferating cells were determined based on CFSE dilution using FlowJo and are reported in graphs. Red dots indicate controls, green indicates HIV peptide antigens, and blue indicates herpesviruses peptide antigens. Experiments were done in duplicates. Intra-individual differences between the highest number of proliferating cells in response to HIV peptide antigen stimulation minus the highest number of proliferating cells in response to herpesviruses peptide antigen stimulation are shown (**Panel C**). The median value is below zero, resulting in a one-sided Wilcoxon p-value of 0.958, indicating that cells do not proliferate more in response to HIV stimulation.
EBV antigens: barf1, bmlf1, bmrf1, brlf1, bzlf1, ebnalp, ebna1, ebna2, ebna3a, ebna3b, ebna3c, lmp1, lmp2. HIV antigens: vpu, rev, vpr, pol1, pol2, pol, gag, vif, env1, env2, tat, nef. HSV-1 antigens: rs-1, ul27. HSV2 antigen: gD2. CMV antigen: pp65.

Supplementary Table 3. Peptide antigen pools used for peptide reactivity assays

	No. of peptides
**EBV**	
PepMix EBV (BARF1)	53
PepMix EBV (BMLF1)	117
PepMix EBV (BMRF1)	99
PepMix EBV (BRLF1)	149
PepMix EBV (BZLF1)	59
PepMix EBV (EBNA-LP)	124
PepMix EBV (EBNA1)	158
PepMix EBV (EBNA2)	119
PepMix EBV (EBNA3a)	234
PepMix EBV (EBNA3b)	279
PepMix EBV (EBNA3c)	265
PepMix EBV (GP350/GP340)	224
PepMix EBV (LMP1)	94
PepMix EBV (LMP2)	122
**HIV***	
HIV-1 Consensus VPU	19
HIV-1 Consensus REV	27
HIV-1 Consensus Vpr	22
HIV-1 PTE Pol-1	240
HIV-1 PTE Pol-2	240
HIV-1 PTE Gag	320
HIV-1 Consensus B Vif	46
HIV-1 PTE Env 1	240
HIV-1 PTE Env 2	240
HIV-1 Consensus B Tat	23
HIV-1 PTE Nef	127
**HSV-1****	
RS-1	321
UL27	223
**CMV***	
pp65 Pool	138
**HSV-2****	
gD2	96

*From NIH repository
**15mer peptides overlapping by 11

Supplementary Table 4. HIV IS gene set enrichment analysis comparing integration sites from *in vitro* acutely HIV-infected primary CD4+ T cells, ART-acute-HIV, and ART-chronic-HIV groups

ART-acute-HIV IS vs. *in vitro* IS	IS from ART-acute-HIV group and MSigDb gene set	IS from ART-acute-HIV group not in MSigDb gene set	IS from *in vitro* dataset and MSigDb gene set	IS from *in vitro* dataset not in MSigDb gene set	OR (ART-acute-HIV IS/*in vitro* IS)	p (Fisher's Exact)	p.holm	fdr.q
KINSEY_TARGETS_OF_EWSR1_FLII_FUSION_UP	60	440	6218	62966	0.7241	0.0227	1	0.6456
VERHAAK_GLIOBLASTOMA_MESENCHYMAL	5	495	1434	67750	2.0954	0.1117	1	0.7012
KOINUMA_TARGETS_OF_SMAD2_OR_SMAD3	23	477	4753	64431	1.5299	0.0499	1	0.693
FISCHER_DREAM_TARGETS	54	446	5268	63916	0.6807	0.0109	1	0.5759
BILBAN_B_CLL_LPL_UP**	9	491	276	68908	0.2185	0.0002	0.1703	0.0853
IIZUKA_LIVER_CANCER_PROGRESSION_G2_G3_UP**	5	495	58	69126	0.0830	0.0000	0.0641	0.0641
MCCOLLUM_GELDANAMYCIN_RESISTANCE_UP**	4	496	47	69137	0.0843	0.0005	0.3448	0.1152
GROSS_HYPOXIA_VIA_ELK3_UP	7	493	1351	67833	1.4026	0.5132	1	1
NUYTTEN_NIPP1_TARGETS_UP	20	480	2959	66225	1.0723	0.9114	1	1

ART-chronic-HIV IS vs. *in vitro* IS	IS from ART-chronic-HIV group and MSigDb gene set	IS from ART-chronic-HIV group not in MSigDb gene set	IS from *in vitro* dataset and MSigDb gene set	IS from *in vitro* dataset not in MSigDb gene set	OR (ART-chronic-HIV IS/*in vitro* IS)	p (Fisher's Exact)	p.Holm	fdr.q
KINSEY_TARGETS_OF_EWSR1_FLII_FUSION_UP	67	453	6218	62966	0.6676	0.0033	1	0.4642
VERHAAK_GLIOBLASTOMA_MESENCHYMAL**	1	519	1434	67750	10.984	0.0004	0.3328	0.1112
KOINUMA_TARGETS_OF_SMAD2_OR_SMAD3	21	499	4753	64431	1.7529	0.0087	1	0.5373
FISCHER_DREAM_TARGETS	56	464	5268	63916	0.6829	0.0099	1	0.5373
BILBAN_B_CLL_LPL_UP	0	520	276	68908	Inf	0.2787	1	0.9159
IIZUKA_LIVER_CANCER_PROGRESSION_G2_G3_UP	0	520	58	69126	Inf	1	1	1
MCCOLLUM_GELDANAMYCIN_RESISTANCE_UP	0	520	47	69137	Inf	1	1	1
GROSS_HYPOXIA_VIA_ELK3_UP**	0	520	1351	67833	Inf	0.0000	0.0523	0.0262
NUYTTEN_NIPP1_TARGETS_UP*	44	476	2959	66225	0.4833	0.0000	0.0194	0.0194

ART-acute-HIV IS vs ART-chronic-HIV IS	IS from ART-acute-HIV group and MSigDb gene set	IS from ART-acute-HIV group not in MSigDb gene set	IS from ART-chronic-HIV group and MSigDb gene set	IS from ART-chronic-HIV group not in MSigDb gene set	OR (ART-acute-HIV/ART-chronic-HIV)	p (Fisher's Exact)	p.holm	fdr.q
KINSEY_TARGETS_OF_EWSR1_FLII_FUSION_UP	60	440	67	453	1.0845	0.7048	1	0.8530
VERHAAK_GLIOBLASTOMA_MESENCHYMAL	5	495	1	519	0.1910	0.1170	0.4683	0.1756
KOINUMA_TARGETS_OF_SMAD2_OR_SMAD3	23	477	21	499	0.8729	0.7582	1	0.8530
FISCHER_DREAM_TARGETS	54	446	56	464	0.9968	1	1	1
BILBAN_B_CLL_LPL_UP*	9	491	0	520	0	0.0015	0.0141	0.0141
IIZUKA_LIVER_CANCER_PROGRESSION_G2_G3_UP**	5	495	0	520	0	0.0280	0.1680	0.0630
MCCOLLUM_GELDANAMYCIN_RESISTANCE_UP	4	496	0	520	0	0.0573	0.2869	0.1033
GROSS_HYPOXIA_VIA_ELK3_UP**	7	493	0	520	0	0.0066	0.0465	0.0199
NUYTTEN_NIPP1_TARGETS_UP*	20	480	44	476	2.2168	0.0041	0.0334	0.0188

*Tier 1 (Holm-adjusted $p < 0.05$) significance

**Tier 2 (FDR Q-value < 0.20 and unadjusted p-value < 0.05) significance

OR: Odds ratio of enrichment; p: p-value (Fisher's exact test); p.holm: Holm adjusted p-value; fdr.q: false discovery rate q-value

Supplementary Table 5. Viral ORF Detection Assay (VODA) primers and probes

Name	Sequence	Target	Reporter	Quencher	Use
probeV1-LTR104-19*	CTGGTAACTAGAGATCCCT	LTR	VIC	MGBNFQ	forward probe
gag-B1	ACCATCAATGAGGAAGCT	gag	6FAM	MGBNFQ	forward probe
env-B2	CCATAGTGCTTCCTGCTGCTCCCAA	env	ABY	QSY	reverse probe
hTFR-exon-Cy5	CCTGAGTTGAACAAAGTGGCACGAGCA	hTFR*	Cy5	BHQ2	forward probe
NEC152*	GCCTCAATAAAGCTTGCCTTGA	LTR			forward primer
5R633alt1	GCTAGAGATTTTCCACACTGACTARA	LTR			reverse primer
5F1372alt1	CAAGCAGCYATGCARATGTT	gag			forward primer
5R1504	TTCCTGCTATRTCACTTCCCCTT	gag			reverse primer
5F7724	GGCAARGAGAAGAGTGGTGCA	env			forward primer
5R7851	GYCTGGCYTGTACCGTCAGC	env			reverse primer
hTFR-exon-F	TGGACACCTATAAGGAACTGATTGAG	hTFR**			forward primer
hTFR-exon-R2	AGTTGGCTGTTGTACCTCTCATAGTC	hTFR**			reverse primer

*From Rouet *et al* (2005)
**hTFR = human transferrin receptor

Supplementary Table 6. HIV-specific multiple displacement amplification (MDA) primers

Primer Name	Primer sequence
MDA.F10s	CACCAAATG*A*A
MDA.F11s	CTGTACCA*G*T
MDA.F12s	TCCATCCT*G*A
MDA.F13s	AACAAATCAG*A*A
MDA.F14s	ATTCCCTAC*A*A
MDA.F15s	CATAGAATG*G*A
MDA.F16s	GCAGGAAG*A*A
MDA.F1s	GACTCGGC*T*T
MDA.F2s	GAGGCTAG*A*A
MDA.F3s	GGAGAGAG*A*T
MDA.F4s	GTATGGGC*A*A
MDA.F5s	CAGAAGAACT*T*A
MDA.F6s	GAAGCTTTA*G*A
MDA.F7s	AACAAAAGTAA*G*A
MDA.F8s	TGGGTAAAA*G*T
MDA.F9s	TACCCATG*T*T
MDA.R10s	TTACTGCTT*T*G
MDA.R11s	TTCTGAAAAA*C*A
MDA.R12s	GTACTGCT*G*T
MDA.R13s	GTCTGTTACT*A*T
MDA.R1s	TGACTGGA*A*A
MDA.R2s	AAGTCTCTC*A*A
MDA.R3s	AACCCAAG*G*A
MDA.R4s	CCACTCTT*C*T
MDA.R5s	CTAATGGTTC*A*A
MDA.R6s	CAGGTCTG*A*A
MDA.R7s	CTCCACAATT*A*A
MDA.R8s	AGTTGAGTT*G*A
MDA.R9s	TGTTCTACC*A*T

*indicates phosphorothioate bond

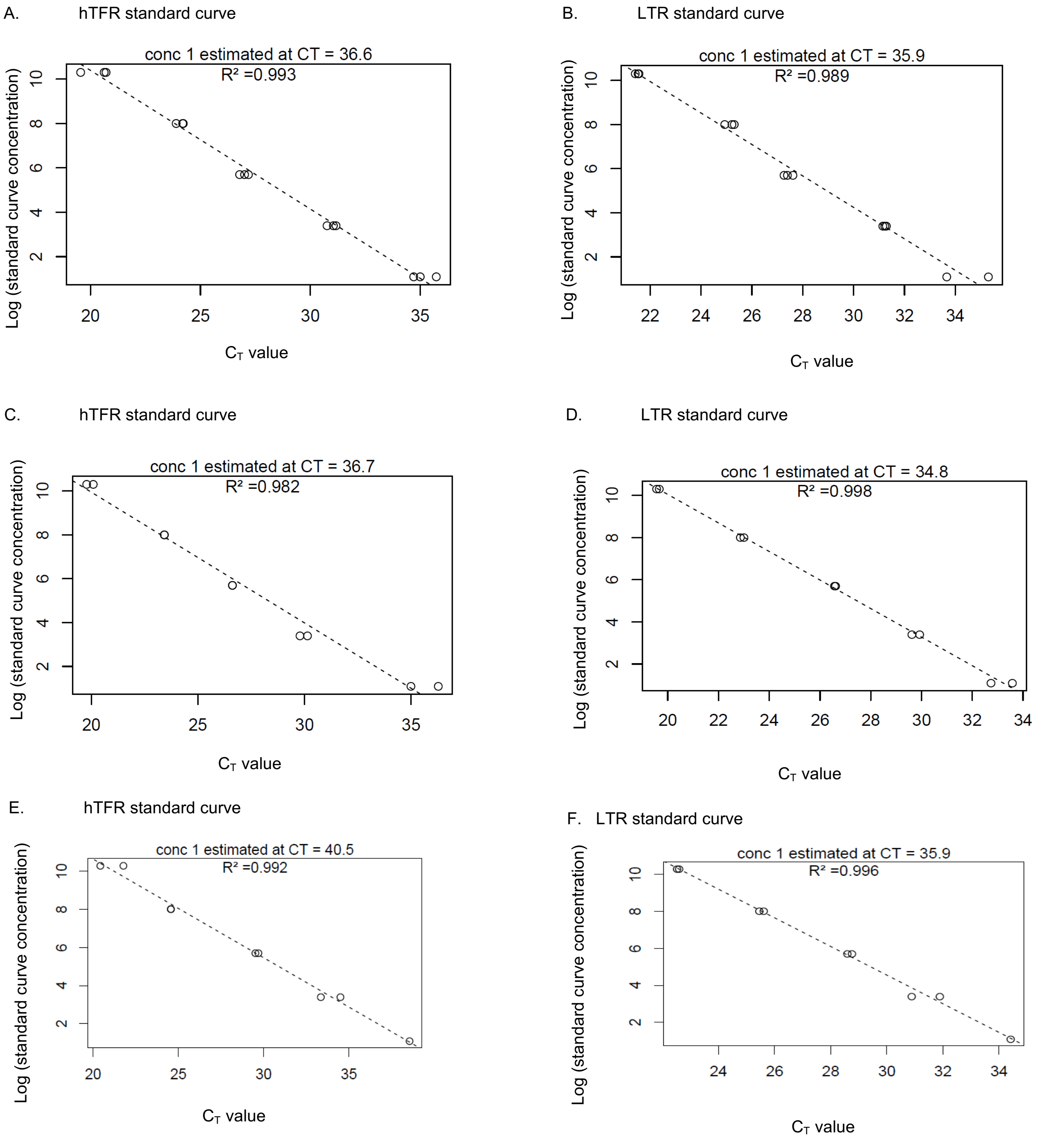

Supplementary Figure 4. Viral ORF Detection Assay (VODA) Standard Curves. HIV DNA was quantified based on fitted standard curves. Variances of the Normally-distributed error of the difference of log concentration estimates for numerator (HIV LTR) and denominator (hTFR) were estimated for each replicate separately. Using a fitted standard curve (for predicting log concentration from C_T) for A) HIV LTR and B) hTFR, we estimated the variance of the mean of the two replicates by considering the variance of each replicate as the sum of the estimated residual variance from the fitted standard curve simple linear regression models for the corresponding qPCR plate. Standard curves are based on data derived from participants 80249, 49467, 51729, 97054, 49021, 59530, 83747 (A, B), 49861 (C, D), 49021 (E, F), 64428 (G, H), and 49467 (I, J).

G. hTFR standard curve

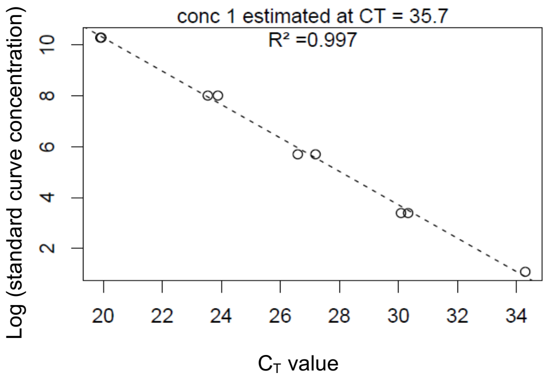

H. LTR standard curve

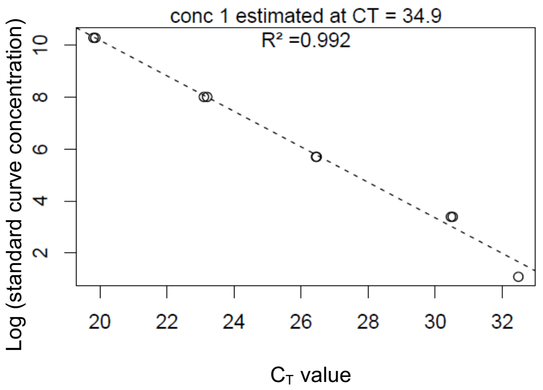

I. hTFR standard curve

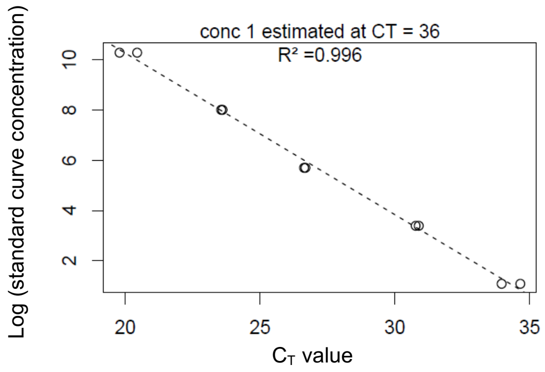

J. LTR standard curve

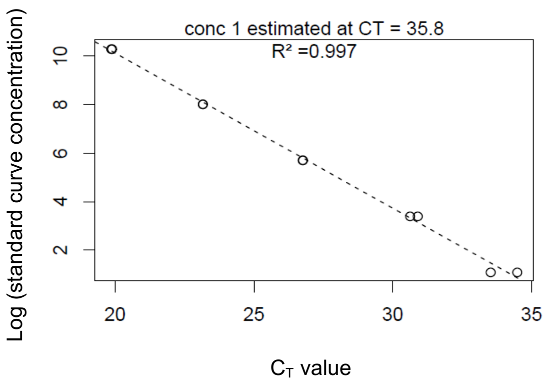

Supplementary Figure 4. con't
